# Hydrogel-based microfluidic device with multiplexed 3D in vitro cell culture

**DOI:** 10.1038/s41598-022-22439-y

**Published:** 2022-10-22

**Authors:** Allison Clancy, Dayi Chen, Joseph Bruns, Jahnavi Nadella, Samuel Stealey, Yanjia Zhang, Aaron Timperman, Silviya P. Zustiak

**Affiliations:** 1 Department of Biomedical Engineering, Saint Louis University, St Louis, MO 63103-2010 USA; 2 Department of Bioengineering, and Biochemistry and Biophysics, University of Pennsylvania, Philadelphia, PA USA

**Keywords:** Cancer microenvironment, Cancer models, Biomaterials, Tissue engineering, Lab-on-a-chip

## Abstract

Microfluidic devices that combine an extracellular matrix environment, cells, and physiologically relevant perfusion, are advantageous as cell culture platforms. We developed a hydrogel-based, microfluidic cell culture platform by loading polyethylene glycol (PEG) hydrogel-encapsulated U87 glioblastoma cells into membrane-capped wells in polydimethyl siloxane (PDMS). The multilayer microfluidic cell culture system combines previously reported design features in a configuration that loads and biomimetically perfuses a 2D array of cell culture chambers. One dimension of the array is fed by a microfluidic concentration gradient generator (MCGG) while the orthogonal dimension provides loading channels that fill rows of cell culture chambers in a separate layer. In contrast to typical tree-like MCGG mixers, a fractional serial dilution of 1, ½, ¼, and 0 of the initial solute concentration is achieved by tailoring the input microchannel widths. Hydrogels are efficiently and reproducibly loaded in all wells and cells are evenly distributed throughout the hydrogel, maintaining > 90% viability for up to 4 days. In a drug screening assay, diffusion of temozolomide and carmustine to hydrogel-encapsulated U87 cells from the perfusion solution is measured, and dose–response curves are generated, demonstrating utility as an in vitro mimic of the glioblastoma microenvironment.

## Introduction

Nearly 97% of drugs developed for oncology interventions fail during clinical trials due to the reliance on inadequate drug models, specifically in vitro 2-dimensional (2D) cell culture models^1^. Though in vitro 2D cell culture models are convenient to use, they do not properly represent the complex tumor microenvironment as cellular behavior is impacted by the flat morphology of the stiff, planar plastic typically used^2,3^. To address the concerns with 2D cell culture models, there has been a shift towards three-dimensional (3D) cancer cell culture models as more physiologically relevant mimics of the tumor microenvironment^4^. Overall, 3D tumor models can take many forms, such as spheroids, organoids, or matrix-based cultures and co-cultures to name a few^5,6^. To add complexity and enhance physiological relevance, 3D tumor models can be combined with microfluidic devices to allow perfusion or mimic vascularization^7^. Microfluidic systems that combine cells, an extracellular matrix, and perfusion are good mimics of the in vivo tumor microenvironment and are low cost and reproducible, enable the use of small culture volumes, and have a controllable design that can be optimized for a desired application^8,9^. Perfusion-based cell culture systems provide nutrients to cells and remove metabolic waste in a manner meant to mimic in vivo mass transport and keep soluble factors near biologic concentrations^10^. In addition, temporal and spatial gradients can be generated within microfluidic systems, further expanding their value as drug screening platforms^8^. However, designing and operating a robust microfluidic perfusion system for 3D culture of adherent mammalian cells is challenging^3^. Challenges could be related to device design, such as choosing the appropriate culture configuration, materials, or microfluidic network fabrication, or purely technical, such as sterilization, cell seeding in the device, optimizing the mass transport and flow shear stresses to maximize cell viability, and avoiding air bubbles in the perfusion channels.

Gradient mixers have been developed for microfluidic systems that can be used to provide on-chip mixing to supply discrete concentrations of soluble factors to cell culture systems that can be used to maintain cells, differentiate stem cells or answer various biological questions^11^. Many of these microfluidic mixers utilize tree-like structures with multiple stages that require complete mixing prior to the end of each stage^8,12,13^, similar to the design used herein. On-chip gradient formation eliminates the possibility for pipetting errors, decreases chances for contamination, and allows for fewer microfluidic inlets that simplify setup compared to microfluidic devices with externally generated gradients^14,15^.

Recognizing the enhanced physiological relevance of perfusion 3D cell culture systems, a growing body of research has focused on microfluidic cell culture devices for drug screening applications with emphasis on liver and cancer tissue models^16,17^. For example, tumor spheroid models were grown in cell culture microwells connected with a concentration gradient generator in microfluidic chips and used in drug screening applications^18,19^. These 3D systems did not incorporate an extracellular matrix mimic and relied on cell capture to fill the cell culture chambers. Utilizing a clever design, a 3D microfluidic cell array incorporated both cancer cells and endothelial cells to emulate the tumor environment^7^. The bottom layer had microchambers with hydrogel-embedded cancer cells, separated from an upper microchannel layer of endothelial cells through a permeable membrane with clustered pores, and the device did not include a concentration gradient generator. The authors then compared drug responses in their system to drug responses in a static 3D culture and noted a delayed response possibly due to the endothelium formed in the top microchannels, serving as a barrier for drug penetration. Another capillary-force based microfluidic device for 2D and 3D cell culture incorporating endothelial and cancer cell layers was developed for drug screening applications, where a gradient could be generated without the need for specialized equipment^20^. Toh et al. developed a 3D microfluidic cell array chip to test drug hepatotoxicity, where hepatocytes were cultured and exposed to gradient drug doses^21^. The 3D microenvironment was generated via collagen and terpolymer coacervation and maintained with a micropillar array. Overall, microfluidic cell culture devices can combine multiple useful features such as an extracellular matrix mimic, cells and cell co-cultures, physiologically relevant perfusion, multiplexed cell culture chambers, and concentration gradient or dilution generators, which make them attractive for applications such as drug screening^22^.

Here, we describe a microfluidic device designed for hydrogel-based 3D cell cultures and showcase its utility with two different hydrogel matrices and building dose–response curves with two chemotherapeutics. The 2D array of cell culture chambers are perfused by a tree-like MCGG that creates a serial fractional dilution that supply dilution ratios of 1, ½, ¼, and 0. The cells and gel precursor solution are easily loaded into the device and the hydrogels form after photoinitiation or Michael-type addition without occlusion of the perfusion channels or pressure buildup that could affect cell viability. Using this microfluidic system, we cultured glioblastoma cells in 3D polyethylene glycol (PEG)-based hydrogels, and measured drug delivery via perfusion and cell viability after treatment with temozolomide (TMZ) and carmustine (BCNU).

## Materials and methods

### Materials

Sylgard™ 184 silicone elastomer kit (referred to as polydimethyl siloxane or PDMS) was obtained from Dow Silicones Corporation (Midland, MI). Polyester (PETE) membrane filters (transparent, 0.2 µm pore size, 12 µm thickness, 2e^6^ pores/cm^2^) were from Sterlitech Corporation (Kent, WA). Pre-cleaned 75 mm × 50 mm × 1 mm plain glass slides were from Corning Incorporated (Corning, NY). INTRAMEDIC polyethylene (PE) tubing (I.D. 1.40 mm, O.D. 1.90 mm; and I.D. 1.14 mm, O.D. 1.57 mm) were from Clay Adams. Silicon (SI) wafers were from University Wafer Inc (Boston, MA). SU-8 2025 photoresist and SU-8 developer (98–100% 1-Methoxy-2-propyl acetate) were from Kayaku Advanced Materials Inc (Westborough, MA). MIL PRF 131k Class 1 foil, 150 µm thick (MarvelSeal^®^ 470) was from Berry Global (Evansville, IN). A1 frame, a thin polymer film (A4 inkjet waterproof film) was from CisInks (South El Monte, CA). Polyethylene glycol diacrylate (PEGDA; 5 kDA), 4-arm poly(ethylene glycol)-acrylate (4-arm PEG-Ac; 10 kDa), and poly(ethylene glycol)-dithiol (PEG-diSH; 3.4 kDa) were from Laysan Bio Inc. (Arab, AL). Phosphate buffered saline (PBS, 10 X, pH 7.4), Trypan Blue (0.4%), fluorescent polystyrene beads (Fluoro-Max™ Fluorescent Red, 542/612 nm, d = 2 µm), and Temozolomide (TMZ) were from Thermo Fisher Scientific (Waltham, MA). Irgacure 2959 was from BASF corporation (Florham Park, NJ). Fetal bovine serum (FBS) and penicillin/streptomycin (P/S) were from Hyclone (Logan, UT). Roswell Park Memorial Institute (RPMI)-1640 medium and 0.05% trypsin/0.02% ethylenedinitrilotetraacetic acid (EDTA) were from Coring (Coring, NY). Acridine orange (AO) and carmustine (BCNU) were from Millipore Sigma (St Louis, MO). Propidium iodide (PI) was from MP Biomedical LLC (Solon, OH). Cell stain 3,3’-dioctafecyloxacarbocyanine perchlorate (DiOC) was from Life Technologies (Carlsbad, CA). Brilliant Blue FCF (CAS Number: 3844-45-9) was purchased as blue food coloring from a local grocery store. Homo sapiens brain glioblastoma U87 cells were from ATCC (Manassas, VA). Glycine–Arginine–Cysteine–Aspartic Acid–Arginine–Glycine–Aspartic Acid–Serine (GRCD-RGDS), Glycine–Arginine–Cysteine–Aspartic Acid–Arginine–Glycine–Aspartic Acid–Serine–FITC (GRCD-RGDS-FITC), and Aspartic Acid–Arginine–Cysteine–Glycine–Valine–Proline–Methionine–Serine–Methionine–Arginine–Glycine–Cysteine–Arginine–Aspartic Acid (DRCG-VPMSMR-GCRD) peptides were from Genic Bio (Shanghai, China).

### Microfluidic device preparation

**Figure 1:**
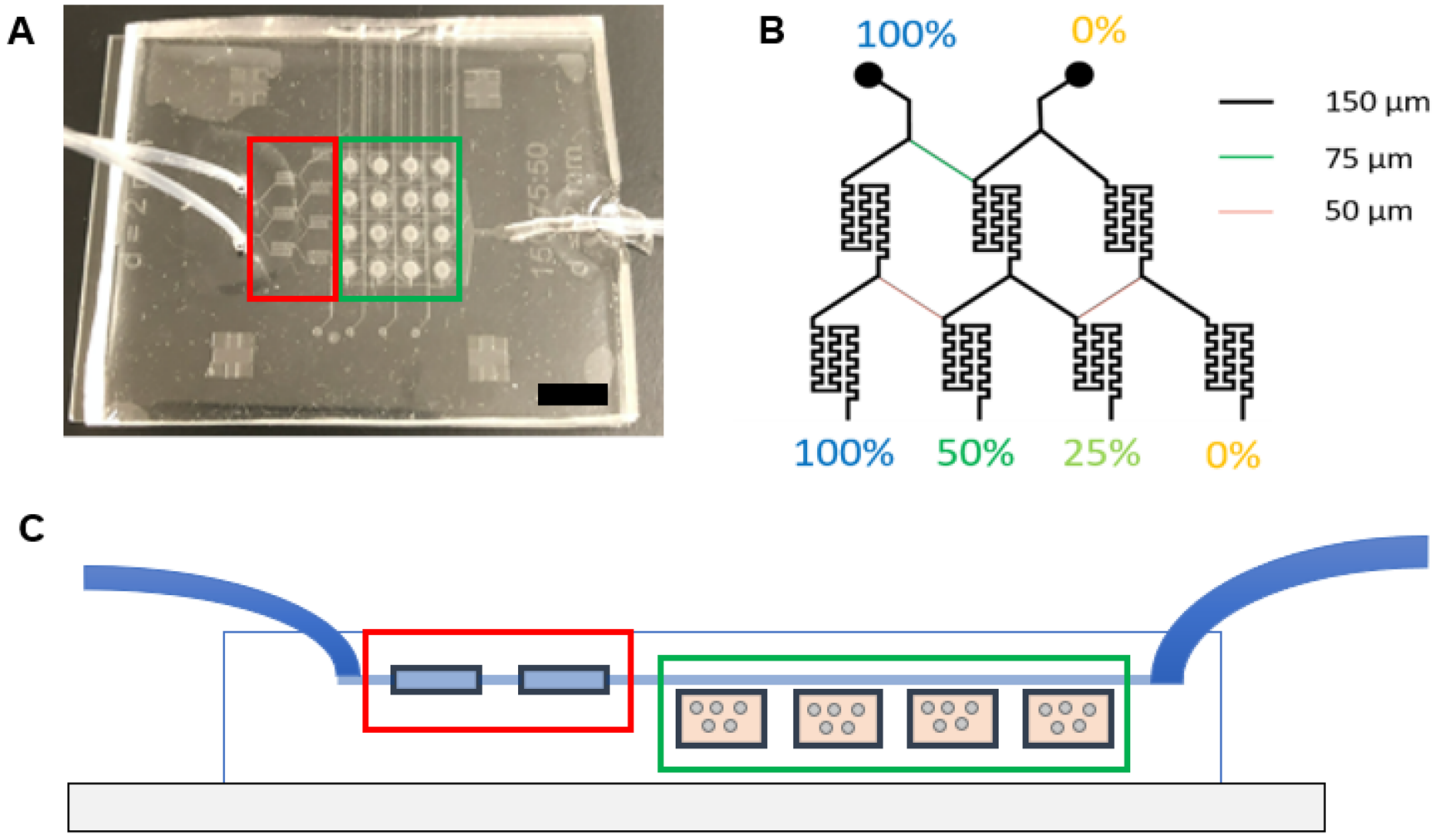
Microfluidic device design. (**A**) Photo of the microfluidic device with the inlet (left-hand side of image) and outlet (right-hand side of image) tubing. Black scale bar represents 1 cm. (**B**) Channel widths of the microchannels and the concentration dilutions generated with these channel widths. (**C**) Side view of the fabricated microfluidic devices displaying the inlet tubes (left), mixing chambers (red), cell culture chambers (green), and outlet tube (right).

The microfluidic device was fabricated by assembling a support glass microscope slide (75 mm × 50 mm × 1 mm), two layers of patterned PDMS (top perfusion layer and a bottom cell culture chambers layer with cell loading channels), sixteen PETE membranes (capping the sixteen cell culture microwells), and three tubes—two inlet tubes (PE tubing; I.D. 1.14 mm, O.D. 1.57 mm) and one outlet tube (PE tubing; I.D. 1.40 mm, O.D. 1.90 mm) (Fig. 1, Supplemental Fig. S1). The top perfusion layer was 3 mm thick and the bottom cell culture microwells layer was 250 µm thick. The height of the perfusion and cell loading channels was 50 µm. A well depth of 250 µm was chosen to be larger than an individual cell while still allowing imaging through the entire sample. Most perfusion channels were 150 µm wide with the key exceptions of 50 µm and 75 µm, which were incorporated into the design to achieve fractional dilution series. These fractional dilution channels were used to produce a concentration gradient of 100%, 50%, 25%, and 0% of initial solute concentration. The cell loading channels on the bottom layer were also 150 µm wide.

Both top and bottom PDMS layers were fabricated via curing PDMS precursor on patterned Si masters (fabricated by standard photolithography SU-8 2025 procedure: https://kayakuam.com/wp-content/uploads/2019/09/SU-82000DataSheet2025thru2075Ver4-3.pdf) at 75 °C overnight. Briefly, a 50 µm thick SU-8 2025 photoresist was spin-coated on a Si wafer at 1700 rpm (SP-100 spinner, BidTec) and soft baked on a hotplate at 65 °C for 3 min and then at 95 °C for 9 min. The patterns on the pre-printed masks were transferred to the photoresist layer through UV radiation (160 mJ/cm^2^ with Flood Exposure Model 60, ABM-USA, Inc., San Josa, California, USA), post-exposure baked (on a hotplate at 65 °C for 6 min and then at 95 °C for 7 min) and developed by a standard process shown in Supplemental Fig. S2. UV radiation crosslinked the exposed SU-8, and SU-8 developer dissolved the uncrosslinked SU-8, leaving only the crosslinked SU-8 patterns.

The bottom cell culture PDMS layer was prepared similarly with a modification to control thickness (Supplemental Fig. S3). To achieve a thickness of 250 µm for the bottom PDMS layer, a 150 µm Al foil frame spacer was used. After pouring PDMS precursor on the Si master and the Al frame, a thin polymer film and a rigid glass slide were put on top. The flexible polymer film was added to avoid the contact of PDMS precursor and the glass slide, otherwise the rigid glass slide could not be removed after PDMS was cured. A 5 lb. weight was added on the top to ensure a uniform thickness of the PDMS layer, and the PDMS was cured at 80 °C overnight. The thickness was measured by an Alpha-Step IQ profilometer (KLA-TENCOR Alpha D500, KLA Corp., Milpitas, California, USA). Note that the PDMS layer was thicker than the spacer as not all excess PDMS was extruded through the edges prior to polymerization due to the high viscosity of the PDMS precursor.

After the PDMS layers were cured, 16 cell chambers (2 mm I.D.), 2 inlets (1.5 mm I.D.) and 1 outlet (2 mm I.D.) were cut with biopsy punches. Sixteen circular PETE membranes (pore size 0.2 µm), cut with 3 mm diameter biopsy punch, were individually placed over each of the 2 mm ID cell culture microwells to separate the two PDMS layers and prevent cells and material in the culture chambers from escaping into the perfusion channels. Individual membrane pieces were chosen as opposed to one piece to enable better adhesion between the two PDMS layers (top and bottom layers of the device), to avoid leakage, and to assure stability over time. Individual pieces also prevent the crosstalk between wells, which could happen with one piece membrane layer. The 3 mm diameter membranes are slightly larger than the 2 mm ID wells, so the edges of the membranes contact the smooth surfaces of the PDMS, adhere to it spontaneously, and stay steady during the following oxygen plasma and alignment. A laboratory corona treatment device (BD-20AC model, Electro-Technic Products, Chicago, IL, USA) was then used to treat the two PDMS surfaces with plasma for 1 min to oxidize them. The two PDMS layers were aligned with four registration marks on the corners by eye. After the two PDMS layers were pressed together with PETE membranes in-between, the glass slide surfaces and PDMS assembly were further oxidized with plasma for 1 min to improve attachment. Inlets and outlet tubing were inserted into the inlets and outlet locations and sealed with uncured PDMS. The devices were heated at 75 °C for at least 2 h to improve sealing and cure the PDMS. A detailed device assembly schematic is shown in Supplemental Fig. S4.

### Mixer performance and dilution analysis

**Figure 2:**
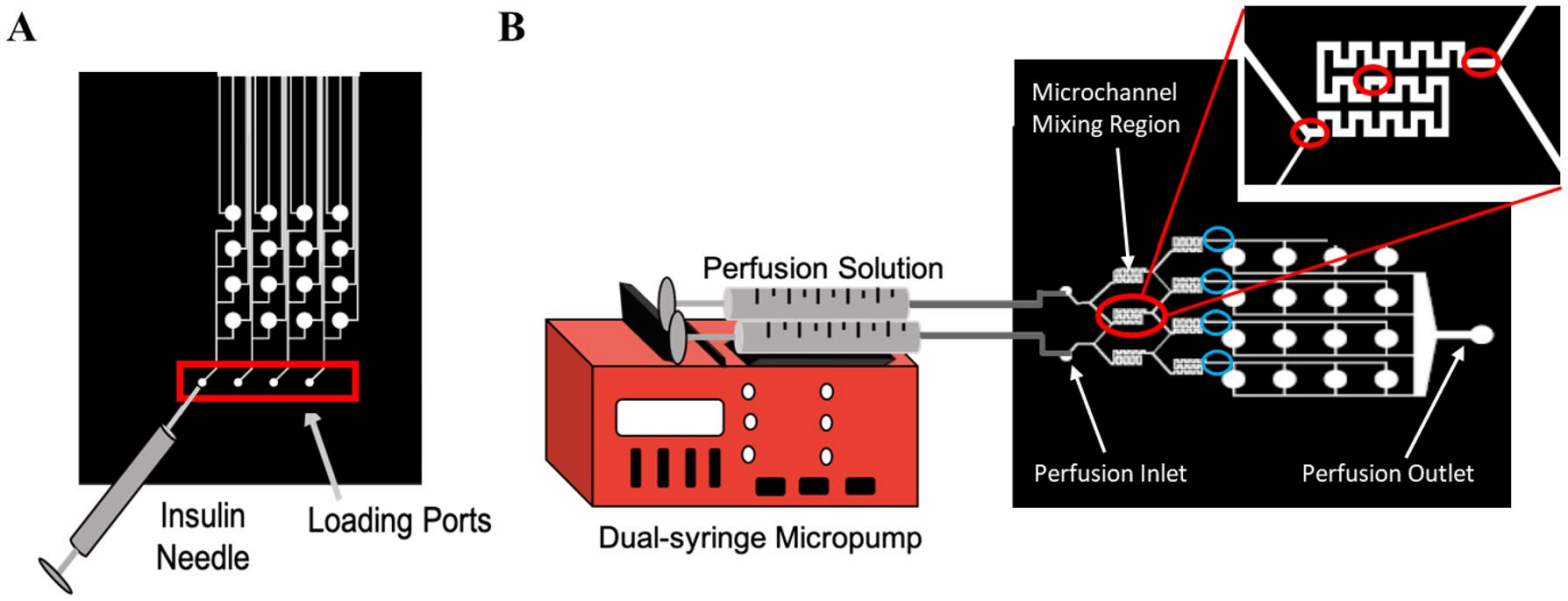
Loading and perfusion set-up. (**A**) Schematic of loading the hydrogel precursor solution into the membrane-capped microwells of the bottom loading layer of the device via the loading ports using an insulin needle. (**B**) Schematic of perfusion set-up in which tubing connects the 3 mL syringes containing the perfusion fluids to the inlet ports of the microfluidic device. Red circles indicate where images were taken within the micromixer for mixing analysis (entrance, middle, and exit). Blue circles indicate where images were taken at the end of the micromixer for concentration gradient analysis.

For perfusion, perfusion fluids were placed in 3 mL syringes in a dual syringe pump (New Era Pump Systems Inc, Farmingdale, NY) so the inputs into both sides of the mixers had the same flow rate. The syringes were connected to the inlet ports of the device with needles (21 G) and tubing (PE tubing I.D. 1.14 mm, O.D. 1.57 mm). Volumetric flow rates tested were 1, 1.5, 2, 5, and 10 µL/min. To study mixing efficiency, a solution of Brilliant Blue FCF (to aid in visualization) was dissolved in PBS for a final concentration of 25 μM (measured via Beer-Lambert Law)^23^ and used in combination with plain PBS to determine the performance of the micromixers. Using an inverted microscope (Zeiss, Axiovert 200 M, Oberkochen Germany) at 5X magnification, images were captured at three regions—entrance, middle, and exit—of the micromixers during perfusion (Fig. 2B). ImageJ software^24^ was then used to split out RGB channels and measure the intensity of the blue RGB channel within the micromixers (as a function of micromixer width) using the ‘Plot Profile’ function. Values were normalized from 0–1 for each set of images, with a gray value of 1 being assigned to the blue color, and a gray value of 0 assigned to the background color of the microfluidic chip. The absolute mixing index (AMI) was calculated as:

1 $$AMI = \frac{\sigma }{< I >}=\frac{\sqrt{\frac{1}{N}{\sum }_{i=1}^{N}({I}_{i}- {< I >}^{2}}}{< I >},$$

where *I*_*i*_ is the local pixel intensity, < *I* > is the mean pixel intensity in the cross section, and *N* I the total number of pixels^25^.

In addition, to determine the concentration gradient generated by the microfluidic device at the set volumetric flow rates, images were also captured at the end of each micromixer and ImageJ software was then used to compare the average intensity of the dye produced by the micromixers.

### Calculation of diffusion coefficient, hydrodynamic radius, and diffusion time of molecules through the width of the mixing channel

Modeling molecules as hard spheres, we used the Stokes–Einstein equation to calculate the diffusion coefficient for molecules:

2 $$D=\frac{kT}{6\pi \mu r},$$

where *D* is the diffusion coefficient, $k$ is the Boltzmann constant, $\mu$ is the dynamic viscosity of water at 37 °C, and $r$ is the hydrodynamic radius of the particle. Assuming that the molecular volume of the particle corresponds to a spherical shape, the hydrodynamic radius of each molecule was calculated as:

3 $$r=\sqrt[3]{\frac{3\frac{MW}{{N}_{A}\rho }}{4\pi }},$$

where *MW* is the molecular weight of the particle (provided by the Royal Society of Chemistry), *N*_*A*_ is Avogadro’s Number, and $\rho$ is the density of the particle (provided by the Royal Society of Chemistry). The time required for a molecule to diffuse across the width of the channel was then calculated as:

4 $$t=\frac{{x}^{2}}{2D},$$

where $t$ is the time required for diffusion to take place and $x$ is the diffusion distance.

### Cell maintenance

Human glioblastoma U87 cells were cultured in RPMI-1640 medium supplemented with 10% FBS and 1% P/S and incubated in a humidified incubator at 37 °C and 5% CO_2_. Cells were passaged by a 5 min exposure to Trypsin/EDTA once an ~ 80% confluency was achieved, and media was changed every 2 days. Cell passages 10–20 were used for experiments. Cells were cultured with DiOC (20 μM) for 24 h to stain all cells prior to all further experimentation.

### Loading of PEG hydrogels in the cell culture chambers of the microfluidic device

Non-degradable, non-adhesive PEGDA hydrogels were prepared by UV photo-polymerization. Briefly, to prepare a stock solution of the photoinitiator, 1% w/v Irgacure 2959 was dissolved in de-ionized water (DI water), sonicated (Branson, Model #2800, 40 kHz) for 90 min and stored at room temperature, protected from the light for up to 2 weeks. PEGDA hydrogel precursor solution (250 µL) of 20% w/v and 0.1% w/v in Irgacure were prepared in PBS and vortexed for 30 s to assure complete mixing. The hydrogel precursor solution was loaded into the cell culture microwells of the microfluidic device through the loading ports by using an insulin needle (Fig. 2A). Each microwell contained approximately 0.2 µL of the hydrogel precursor solution; hence 3.14 µL of solution were needed to fill all 16 of the microwells. The excess amount of precursor solution was necessary to ensure that the solution reached the microwells instead of remaining within the loading channels. Relief channels were incorporated into the design of the loading layer of the device to enable the excess precursor solution to exit the device instead of pushing against the membrane capping the microwells. The hydrogels were then polymerized under UV lamp (4.81 mW cm^2^, 1 W, 365 nm; Blak-Ray^®^ XX-15L UV bench lamp, UVP, Upland, CA) for 10 min^26^. These gels are referred to as PEGDA throughout.

Degradable, adhesive 4-arm PEG-Ac hydrogels were prepared by Michael-type addition. First, 4-arm PEG-RGDS was prepared following a previously developed protocol^27^. We modified on average one of the acrylate groups of each 4-arm PEG-Ac with RGDS (80% modification efficiency) and stored the lyophilized product under argon in a desiccated container at − 20 °C for up to 6 months. Hydrogels were then formed using a mixture of 4-arm PEG-Ac and 4-arm PEG-RGDS (0.8 mM in RGDS) and a 50:50 crosslinker mixture of PEG-diSH and an enzymatically degradable peptide crosslinker DRCG-VPMS↑MR-GCRD. For all gels, 20% w/v stock solutions of 4-arm PEG-Ac, PEG-diSH, and 4-arm PEG-RGDS were prepared in TEA buffer pH 8 immediately prior to use. Stock solutions were mixed and the peptide crosslinker was added as powder to give a hydrogel precursor solution with a 1:1 acrylate to thiol molar ratio and a final polymer concentration of 10% w/v and a final peptide crosslinker concentration of 7.8 mM. The hydrogel precursor solution was mixed via pipetting for 30 s, injected in the microfluidic device as described above, and allowed to gel for 20 min in a humidified incubator at 37 °C and 5% CO_2_. These gels are referred to as 4-arm PEG-Ac throughout.

To assess the reproducibility of hydrogel and cell loading into the cell culture microwells of the microfluidic device, DiOC-stained U87 cells were added to the PEGDA hydrogel precursor solution at 10^6^ cells/mL. The precursor solution was then loaded into the microwells using an insulin needle and gelled under UV as described above. Images of each well were taken using an inverted fluorescence microscope and ImageJ was used to count the number of ells within each well. The distribution of the fluorescently labelled cells within the hydrogel was imaged using a confocal microscope (Leica Confocal SP8, Leica Microsystems Inc, Buffalo Grove, IL) at × 10 magnification and processed using Fiji software (free download http://fiji.sc). For confocal imaging the hydrogel was also rendered fluorescent by covalently incorporating a fluorescent GRCD-RGDS-FITC peptide as described by us previously^27^.

### Drug screening

A hydrogel precursor solution containing 20% w/v PEGDA, 0.1% v/v Irgacure, and 10^6^ cells/mL (DiOC-stained U87 cells) was injected and polymerized in the microfluidic device cell culture microwells as described above to prepare the non-degradable, non-adhesive PEGDA hydrogels. Immediately after gelation, RPMI media supplemented with 10% FBS and 1% P/S was perfused through the device for 48 h. At 48 h the perfusion media was replaced with supplemented media containing 0.2 μg/mL PI (staining nuclei of dead cells) in both inlets and 2 mM TMZ or 10 µM BCNU in one inlet and perfused through the device for 48 h. Similarly, degradable, adhesive 4-arm PEG-Ac hydrogels containing 10^6^ cells/mL of DiOC-stained U87 cells, were prepared as described above, cultured for 48 h and exposed to TMZ for 48 h. Encapsulated cells were imaged using Axiovert 200 M inverted fluorescence microscope at × 10 magnification. Cell viability was calculated as:

5 $$Cell \,viability \left(\%\right)= \frac{{N}_{L}}{{N}_{L}+{N}_{D}}\times 100,$$

where *N*_*L*_ represents the number of live cells and *N*_*D*_ represents the number of dead cells.

### Fluorescence correlation spectroscopy

Fluorescence correlation spectroscopy (FCS; Zeiss LSM 510 Fluorescence Microscope, Zeiss, Germany) was used to measure in situ diffusivity of a model fluorophore (Atto 655) in a 20% w/v PEGDA hydrogel slab. Hydrogels were formed as described above, with the modification that Atto 655 (0.5 nM) was added to the hydrogel precursor solutions. For FCS measurements, hydrogels (150 µL) were prepared in an 8 chamber coverglass with #1 German borosilicate bottom. Hydrogels were then soaked overnight in DMEM medium without phenol red and containing 0.5 nM Atto 655 to avoid concentration-driven diffusion of Atto 655 from the hydrogel into the surrounding media. Atto 655 (0.5 nM) in deionized water was also used to calibrate the confocal volume of the FCS instrument. A 633 nm ps pulsed laser was used for six measurements of 300 s for each sample location. An autocorrelation function *G(τ)* was obtained for each measurement:

6 $$G\left(\tau \right)=\left[\frac{1}{N}\frac{1}{\left[1+\left(\frac{\tau }{{\tau }_{D}}\right)\right]}\frac{1}{{\left[1+p\left(\frac{\tau }{{\tau }_{D}}\right)\right]}^{0.5}}\right]\left[1+\frac{T}{1-T}{e}^{\frac{-\tau }{{\tau }_{T}}}\right],$$

where *N* is the number of fluorescent particles, *p* = *r*_*o*_*/z*_*o*_ is an instrumental constant, *r*_*o*_ is the radius and *z*_*o*_ is the axial length of the focused laser beam spot, *τ*_*d*_ is the solute diffusion time, *T* is triplet state amplitude, and *τ*_*T*_ is the triplet lifetime. The autocorrelation function was fit using a Triplet model to account for the possible excitation of molecular triplet states at higher laser intensities. Lastly, the autocorrelation function was normalized as follows:

7 $$Normalized \,G\left(\tau \right)=G({\tau }_{D})/G({\tau }_{0}),$$

where *G(τ*_*D*_*)* is the value of Eq. (6) at each time point and *G*(*τ*_0_) is the value of Eq. (6) at the initial time point. The effective tracer diffusion coefficient for each protein in solution was calculated from *τ*_*D*_ as:

8 $${D}_{FCS}={\left({r}_{0}\right)}^{2}/4{\tau }_{D}.$$

Assuming the diffusivities of TMZ and Atto 655 were similar due to their relatively similar molecular weights (194 g/mol and 887 g/mol, respectively), we then modeled the anticipated TMZ concentration in the gels as a function of gel depth and time. TMZ concentration was calculated via Fick’s second law of diffusion for 1-D geometry (a thin slab) with the following boundary conditions: *c*(*x* = 0) = 2 mM TMZ and *c*(*x* = ∞) = 0 mM TMZ at *t* = 0 h, where *c* is the TMZ concentration, *x* is the gel location and *t* is time:

9 $$c\left(x,t\right)=2 {\text{mM}}\times [1-\text{erf}\left(x/2sqrt(Dt)\right)].$$

### Statistical analysis

All data are presented as mean values (± SD) determined from 3 to 4 independent experiments. Statistical analysis for concentration gradient analysis and loading reproducibility was performed using one-way ANOVA with Holm–Sidak or Dunn’s multiple comparison tests using GraphPad Prism 6^®^. Statistical analysis for the slope of the intensity profile vs volumetric flow rate was performed using linear-regression analysis using GraphPad Prism 6^®^. A *p* < 0.05 was considered statistically significant.

## Results and discussion

### Device design, advantages and limitations

Here we designed a microfluidic device which allowed us to load hydrogel-encapsulated cells into membrane-capped cell culture microwells to be perfused with fluids containing desired biomolecules or drugs of varying concentrations (Fig. 1, Supplemental Fig. S1). The device has a combination of several features that make it a good choice for 3D hydrogel-based perfusion cell culture: (i) it is an enclosed structure with cell loading channels; (ii) there is a 2D array of cell culture chambers capped by a membrane to prevent contamination or cell escape into the perfusion layers; (iii) cells are loaded within a hydrogel for biomimetic 3D cell culture; (iv) the inverted design allows for imaging from the bottom; and (v) the MCGG uses channel width as opposed to length to vary mixing ratios.

The microfluidic device was constructed with PDMS, which is commonly used in microfluidic applications as it is simple to fabricate, inert, non-cytotoxic, gas permeable, and suitable for fluorescence imaging^3,28,29^. Importantly for our design, PDMS is complaint and enabled the use of an insulin needle for loading cells and gels in the microwells. The microfluidic system consisted of two microfluidic channel layers, separated by membranes to seal the bottom cell-loading channels. The top perfusion layer (Figs. 1, 2B) contains the serpentine micromixers leading to the perfusion channels that provide flow over the membrane to feed the cells in the culture chambers below. The two-stage micromixers have feed channel widths of 150/75/150/150 μm upstream of the first stage and 150/50/150/50/150 μm upstream of the second stage to produce the fractional concentration dilution series of 1:½:¼:0 (Fig. 1B). The perfusion channels fluidically address the cell culture chambers in parallel to avoid crosstalk. The device provides stable operation over several days, consuming < 1.5 mL/day and perfuses the cells with fresh media to avoid cytotoxicity. At the volumetric flowrate of 1 μL/min, the linear velocity is ~ 1.1 mm/s within the feed channel to the perfusion layer above the cell culture chamber, which is similar to the in vivo flowrate through capillaries^30^. For example, Giulitti et al. demonstrated that if perfusion is too slow (e.g. 0.6 µL/h), downstream cells can experience cytotoxicity from the heterogeneous distribution of nutrients (higher concentration upstream) and waste (higher concentration downstream)^31^. Higher flowrates (e.g. 30–60 μL/h, similar to the 1 μL/min used in this study) result in more homogenous cell culture within the entire microfluidic device. Based on device design, flow rates that are too high (e.g. 40 μL/min) could also result in incomplete mixing or shear stress-induced cytotoxicity^32^.

Microchannels for loading cells into culture chambers were incorporated into the bottom layer. Each row of cell culture chambers is loaded from a single inlet. If the same cell containing solution is loaded into each inlet, four biological replicates are created as performed in this study. Loading is most consistent when each cell culture chamber has the same length of the microchannel pass through it (the sum of the channel length from the inlet to the microwell and from the microwell to the exit) to allow microwells to be filled in parallel simultaneously (Fig. 2A). The channels from each cell culture microwell to the exit acted as a sample relief channel to ease the loading process and reduce pressure required while loading the hydrogel precursor solution into the microwells. The relief channels prevent the hydrogel-encapsulated cells from escaping into the perfusion layer and blocking perfusion upon gelation under excess loading pressure. Each cell culture well was capped with a PETE membrane (pore size of 0.2 µm), which enabled the exchange of nutrients and waste between the cells and the perfusing fluids but was restrictive for large particles, bacteria, and cells, assuring that no contamination could occur due to perfusion. Lastly, the cells contained within the gel matrix were shielded from hydrodynamic shear stress of the circulating perfusion fluids for improved recapitulation of the in vivo system. The location of the culture chambers in relation to the perfusion stream is important as cells located directly in the flow-path are subjected to hydrodynamic shear stress, which can affect the cell morphology, cytoskeleton organization, proliferation, cellular signaling pathways, and gene and protein expression^3^.

### Tree-like microfluidic concentration gradient generator

**Figure 3:**
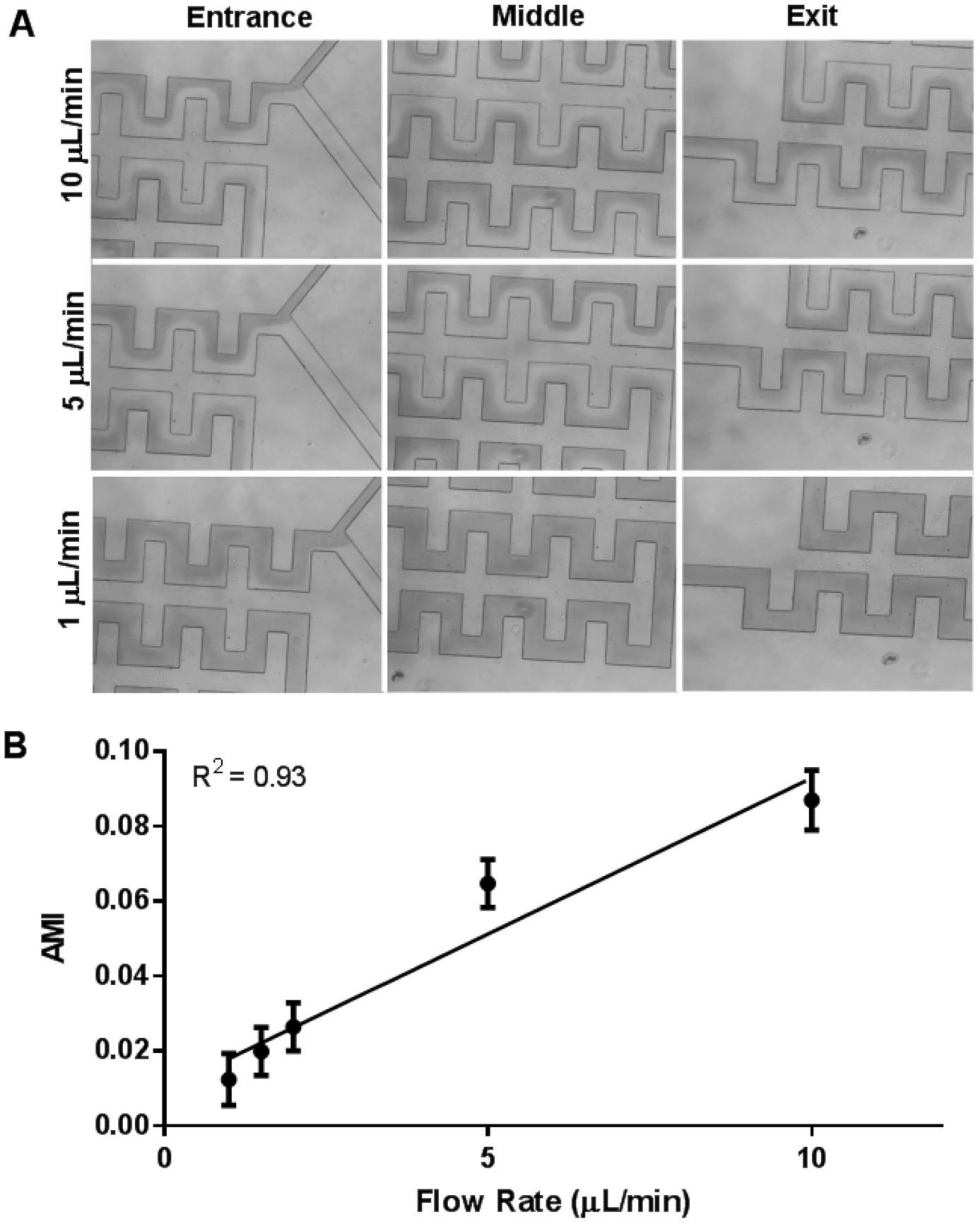
Mixing efficiency. (**A**) Representative microscopy images showing progression of the mixing of PBS and Brilliant Blue FCF in PBS at the entrance, middle, and exit regions of interest within the mixing channels at 10, 5, 2, 1.5, and 1 µL/min. (**B**) The slope of the intensity profile to indicate mixing efficiency at the exit region of the mixing channel as a function of perfusion volumetric flow rate. Calculated absolute mixing index (AMI) at the exit region of the mixing channel as a function of perfusion volumetric flow rate. An AMI value of zero indicates complete mixing.

Here, in contrast to a typical MCGG, a fractional serial dilution is achieved by varying the widths of the feed channels. Diffusive-based mixers have a maximum velocity at which complete mixing is achieved, hence, varying the feed channels width as opposed to length allows for a more compact mixing structure. Previously, a non-dimensional parameter has been created to determine the required mixer length for various geometries^33^. The maximum velocity for gradient mixing is experimentally measured using a microscope in our squared corner 18.5 mm long serpentine channels as shown in Fig. 2B. Images that measure mixing at the entrance, middle, and end of the channel at flowrates of 1, 5, and 10 μL/min are shown (Fig. 3A). In total, five volumetric flow rates (1, 1.5, 2, 5, and 10 µL/min) were used to determine the extent of mixing at the exit of the mixing channel. Complete mixing is indicated by a homogenous solution concentration across the microchannel width, which yields a slope of zero for a plot of intensity vs. distance. In Fig. 3B an AMI of 0 indicates complete mixing. At a flow rate of 1 μL/min, the AMI value was calculated to be 0.012 at a flowrate of 1 μL/min. At greater flowrates, the AMI increased, indicating incomplete mixing, where the extent of mixing decreases as the flowrate increases. Overall, mixing is linearly dependent on the volumetric flow rate (R^2^ = 0.93). Given this fixed mixing length on the device, the dependence of the mixing on the flowrate was measured to ensure adequate mixing at 1 μL/min as shown in Fig. 3B.

Since mixing depends on diffusion, it is expected that mixing efficiency as a function of flow rate depends on molecule size, where *MW* and size determine molecular diffusivity^34^. We used Brilliant Blue FCF, that has a molecular weight (*MW*) of 792.9 g/mol to characterize the mixing, which is larger than most chemotherapeutic agents as shown in Table 1. Complete mixing of Brilliant Blue FCF dissolved in PBS was achieved at 1 µL/min and the time required for diffusion was 34.0 s. The diffusion coefficient and time required for diffusion across the width of the channel was also calculated for several FDA-approved chemotherapeutics: temozolomide, paclitaxel, doxorubicin, carmustine, and lomustine. For example, according to our calculations, temozolomide, which has a smaller diameter and higher diffusivity than Brilliant Blue FCF, would diffuse across the channel width almost 2-times faster. This result implies that a volumetric flow rate greater than 1 µL/min could still lead to complete mixing. On the other hand, paclitaxel, which is similar in size and diffusivity to Brilliant Blue FCF, should require similar time to diffuse across the channel width (31.2 s). Hence, a volumetric flow rate of 1 µL/min should be used to achieve complete mixing. Overall, since all molecules considered were smaller than the Brilliant Blue FCF used, we anticipate that the physiologic 1 µL/min volumetric flow rate would be sufficient to achieve complete mixing for most experiments aiming to produce a concentration dilution of small molecules, such as chemotherapeutics.

**Table 1:** The diffusion coefficients and time required for diffusion of particles of varying sizes calculated using their respective molecular weights and density.

	MW (g/mol)	(g/cm^3^)	r (m)	D_0_ (m^2^/s)	t (s)
Brilliant Blue FCF	792.9	1.0	6.80E−10	3.31E−10	34.0
Temozolomide	194.2	2.0	3.38E−10	6.66E−10	16.9
Paclitaxel	853.9	1.4	6.23E−10	3.61E−10	31.2
Doxorubicin	543.5	1.6	5.13E−10	4.39E−10	25.6
Carmustine	214.1	1.7	3.69E−10	6.09E−10	18.5
Lomustine	233.7	1.4	4.05E−10	5.56E−10	20.2

**Figure 4:**
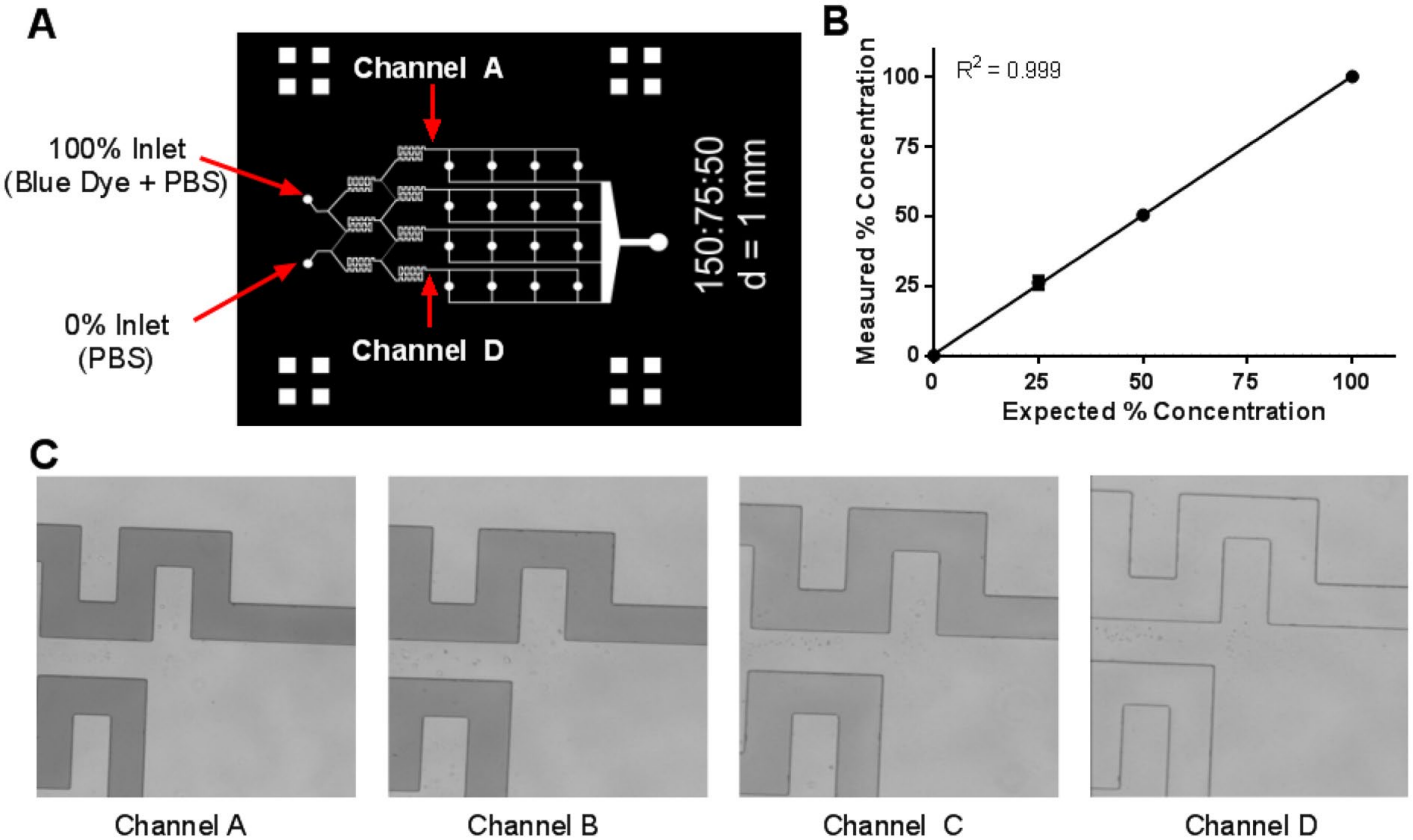
Gradient generation. (**A**) Schematic indicating channels A–D, where the generated concentration gradient was measured. (**B**) Measured concentrations at 1 µL/min flow rate, where channel A contains 100% of the initial concentration of Brilliant Blue FCF and channel D contains 0% Brilliant Blue FCF. (**C**) Representative images of the concentration gradient in each channel at 1 µL/min flow rate.

The concentrations generated by the MCGG were measured by perfusing 25 μM of Brilliant Blue FCF in PBS in the ‘100% inlet’ and PBS in the ‘0% inlet’ at 1 µL/min (Fig. 4A), which produced concentrations of 100%:51%:26%:0% (Fig. 4B), similar to the expected dilution of 100%, 50%, 25%, and 0%. The dilution fractions were interpolated using the intensities of the 100% and 0% microchannels before splitting (Fig. 4C). The intensity in channel A (closest to the inlet containing Brilliant Blue FCF in PBS) was set to 100%, while the inlet containing PBS (Channel D) was set to 0%. The experimentally generated concentration dilution was like the predicted theoretical concentration dilution (Fig. 1B), with an R^2^ value of 0.99, further demonstrating that complete mixing of the two perfusion fluids was achieved at 1 µL/min.

### Cell loading reproducibility

**Figure 5:**
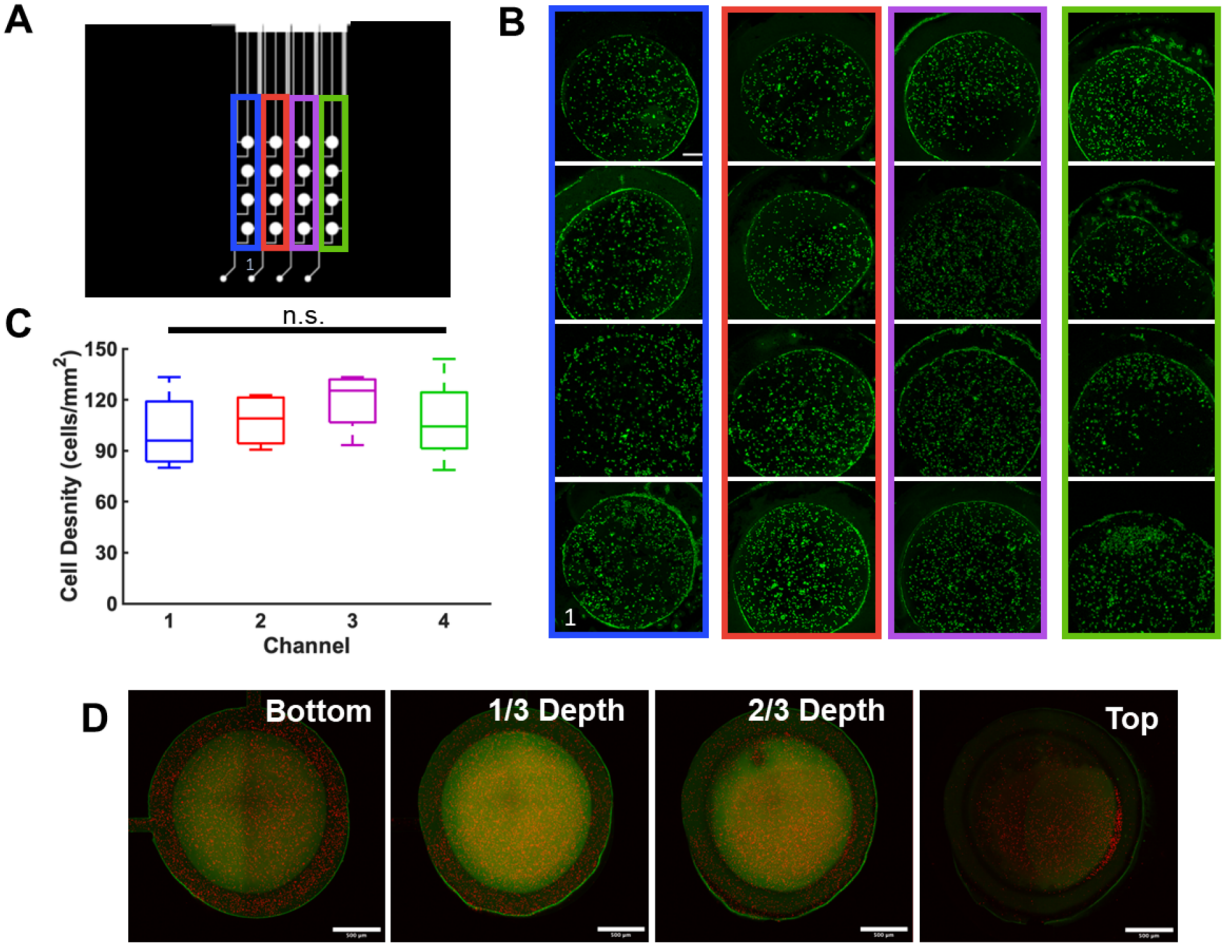
Cell loading reproducibility. (**A**) Device schematic of loading ports and channels and the cell culture microwells, where each column of cell culture chambers is color coded, starting with column 1 in blue. (**B**) Fluorescent images of the cell culture chambers loaded with hydrogels with entrapped U87 cells stained with DiOC. Scale bar = 200 µm. (**C**) Density of cells within the cell culture microwells of each column. (**D**) Images of the bottom, one-third, two-thirds, and the top of a hydrogel when loaded in a cell culture microwell. The hydrogels are labelled with green fluorescence via tethering of FITC-modified ligands to the 4-arm PEG-Ac. Scale bar = 500 µm.

The loading reproducibility of hydrogels into the microwells, as well as the similarity of loading across all wells within the device was characterized. Consistent loading of the cells is facilitated by the flow-through design of the loading channels. The number density of the cells and their location should be stochastic with shot-noise being the main source of loading variability without a source of systematic bias. With the flow-through design of the loading channels, the concentration of the cells should be the same as it is in the original homogenously mixed solution in the absence of cell accumulation or depletion. To measure loading reproducibility, PEGDA hydrogels with encapsulated DiOC-stained cells were loaded into the microwells using an insulin needle and then exposed to UV light for gelation. As all four microwells within a column were loaded simultaneously (Fig. 5A), the average density of the cells within the wells of a column were compared (Fig. 5B). Our results indicate that the loading reproducibility was similar among the 4 columns as there was no significant difference in the median cell density for each column (Fig. 5C).

In a separate experiment, we used fluorescent PEG (green) loaded with red fluorescent beads, to determine whether the hydrogel occupied the whole well evenly and whether the beads were evenly distributed through the depth of the hydrogel. Images captured throughout the depth of the well indicated that the hydrogel filled the microwells and the fluorescent beads remained well-distributed throughout the entirety of the hydrogel (Fig. 5D). However, the green gel appears not polymerized near the PDMS wall and the membrane, which are regions with high oxygen exposure during the polymerization. It is known that oxygen inhibits UV polymerization^35^ and that it has an elevated concentration in PDMS^36^. Also, small hydrophobic molecules such the photoinitiator used here easily diffuse into PDMS, possibly depleting their concentration in the gel precursor solution, which could also explain the ‘halo’ seen in the proximity of the PDMS walls. This shape of hydrogel structure increases the surface area through which solute exchange can occur but does not alter the total amount of solute that can be exchanged, which is determined by the volumetric flow through the perfusion layer. Dependent on the exact application, this hydrogel shape can be either beneficial or detrimental. Future work will investigate methods to lower the oxygen concentration in the PDMS prior to polymerization using non-cytotoxic methods. We have developed methods to fully polymerize hydrogel plugs within PDMS microchannels^37^ but have not tested the cytotoxicity of this protocol. Note that when a 4-arm PEG-Ac gel, which polymerizes via Michael-type addition, was loaded in the cell culture wells, it polymerized completely, including in the immediate vicinity of the PDMS walls (Supplemental Fig. S5) because polymerization via Michael-type addition is not affected by oxygen and does not depend on a small molecule initiator. This data also showed that a variety of hydrogel matrices can be accommodated with the current device.

One challenge of loading cells in microfluidic devices in general is eliminating air bubbles that can be trapped in the microwells during loading. To avoid air bubbles, others have first loaded the system with water and then degassed it^38^. We chose to load the device dry (not pre-filled with water) to eliminate dilution of the gel precursor solution with water. Air bubbles are a routine occurrence in microfluidic devices^39,40^. While we show that in our system bubbles did not negatively impact cell viability (see Supplemental Fig. S7), they could create differences between replicate cell culture chambers. An improvement that could be considered in the future would be changing the geometry of the microchannels within the device^39^ to remove the sharp corner between the loading channels and the reservoirs. The current sharp corners present in both the loading and perfusion layers could trap air, as the angle of the corner is less than that of the contact angle of the filling liquid, creating a lower wettability situation.

### Drug penetration and cell drug responsiveness

First, we determined the time it would take for a small molecule (e.g. chemotherapeutic) being perfused to diffuse into the hydrogel and reach the same concentration as the perfusion channel. To measure this diffusion, we used a fluorophore Atto 655 (887 g/mol) to model the diffusivity of a small molecule drug such as TMZ (194 g/mol) or BCNU (214 g/mol), which allowed us to measure diffusion coefficient in situ and in real time using FCS (Supplemental Fig. S6). Diffusion coefficients for the fluorophore Atto 655 in media, as well as in PEGDA hydrogels were measured as 4.25 × 10^–6^ and 2.55 × 10^–6^ cm^2^/s, respectively. As expected, the diffusion coefficient in media was significantly higher than that in the hydrogel, which could be explained by physical obstruction, as shown by us previously^41^. With the measured diffusion coefficients, we then used Fick’s law of diffusion to determine whether TMZ or BCNU were able to penetrate the hydrogel and how long it would take for the drugs to reach the desired concentration inside the gel. We modeled TMZ concentration (at 2 mM initial concentration) and BCNU concentration (at 10 µM initial concentration) as a function of time (up to 48 h) at a hydrogel depth of 250 μm (the thickness of the gel inside the microwell). Drug concentration was found to be > 1.9 mM within 1 h and > 1.95 mM within 4 h upon TMZ addition and > 9.5 µM within 1 h upon BCNU addition, indicating that for 48 h of exposure, all cells should be equally exposed to both drugs.

We next tested U87 cell susceptibility to two different drugs, namely TMZ and BCNU, in two different PEG hydrogels. TMZ is the current standard of care and BCNU is used as an adjuvant or in combination with TMZ. Both drugs are approved for the treatment of glioblastoma and both are alkylating agents known to induce cell cycle arrest and apoptosis^42^. The two gels were PEGDA and the enzymatically degradable, RGD-modified 4-arm PEG-Ac gel. The PEGDA gel, formed via UV photopolymerization, is non-degradable and inert, hence cells were not expected to interact with it. It served as scaffolding only and cells encapsulated in it for up to 4 days retained high viability (> 90%) (Supplemental Fig. S7A) but remained round (Supplemental Fig. S7B). The 4-arm PEG-Ac gel is degradable and cell adhesive, and cells were able to interact with the hydrogel and remodel it over time as evidenced by cell spreading at day 4 and cell viability > 95% (Supplemental Fig. S7).

**Figure 6:**
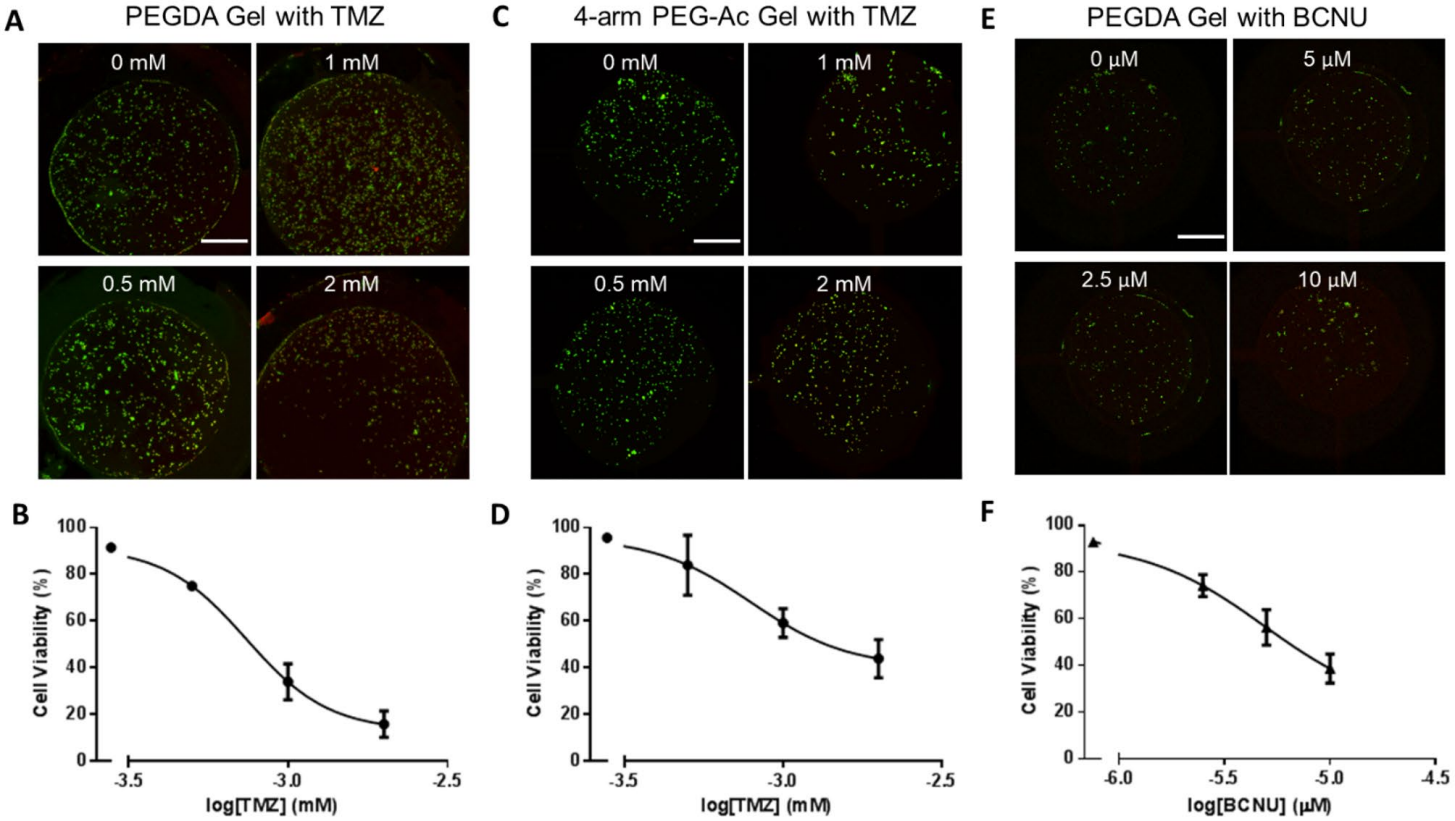
Drug responsiveness. (**A**) Representative U87 cell live/dead images upon TMZ treatment, and (**B**) cell viability as a function of TMZ concentration (n = 3) for cells seeded in a non-degradable, non-adhesive PEGDA gel. (**C**) Representative U87 cell live/dead images upon TMZ treatment, and (**D**) cell viability as a function of TMZ concentration (n = 3) for cells seeded in a degradable, adhesive 4-arm PEG-Ac gel. (**E**) Representative U87 cell live/dead images upon BCNU treatment, and (**F**) cell viability as a function of BCNU concentration (n = 3) for cells seeded in a non-degradable, non-adhesive PEGDA gel. All cells were stained with DiOC (green) and dead cells were stained with PI (red). Scale bar is 200 µm.

Upon treatment with 0, 0.5, 1, and 2 mM TMZ, PEGDA-encapsulated U87 cells were found to have viabilities of 86.4 ± 1.9%, 75.0 ± 1.2%, 34.0 ± 7.7%, and 15.8 ± 5.7%, respectively (Fig. 6A,B). The EC_50_ (effective concentration needed to kill 50% of the cells) was estimated to be ~ 0.61 mM TMZ as calculated by a sigmoidal curve fit (R^2^ = 0.983). Cells were less susceptible to TMZ when encapsulated in the degradable, adhesive 4-arm PEG-Ac hydrogel (Fig. 6C,D). Cell viability was 95.6 ± 1.2%, 83.9 ± 12.9%, 59.1 ± 6.1%, and 44.0 ± 8.2% for 0, 0.5, 1, and 2 mM TMZ, respectively and the EC_50_ was ~ 1.8 mM (R^2^ = 0.901). This result was not surprising as integrin binding has been previously implicated in drug resistance of glioblastoma cells^43–45^, and we have shown similar results previously for glioblastoma spheroids^46^. The EC_50_ for U87 cells encapsulated in a PEGDA hydrogel and treated with BCNU was ~ 8.2 µM (R^2^ = 0.952) (Fig. 6E,F). As expected for all conditions, the estimated EC_50_ was higher compared to 2D monolayer culture, but similar to what has been reported by others for hydrogel-encapsulated GBM cells^2^. Dead cells were visible throughout the gel after 48 h of exposure for all conditions, suggesting that the microenvironment within the gels was homogeneous (Fig. 6A,C,E).

Our results suggest that the device could be used for testing the effect of cell–matrix interactions on cell responsiveness to soluble factors, such as chemotherapeutics, where the therapeutic could readily and homogenously penetrate the cell-seeded hydrogels. While not explored here, the device could be adapted for use with different cells and cell co-cultures and screen their responses to different soluble molecules. Since the design includes hydrogel-encapsulated cells, it could be used to study the effect of cell–matrix and cell–cell interactions on cell responsiveness to soluble factors, such as therapeutics in a physiologically relevant environment that includes perfusion and enables the formation of concentration dilutions.

## Conclusions

In this study, we designed a drug screening microfluidic device incorporating microwells filled with hydrogel-encapsulated cells. The device featured relief channels to allow for seamless hydrogel loading and membrane-capped wells to prevent contamination and perfusion channel blockage. We characterized the mixing efficiency of the device using plain PBS and Brilliant Blue FCF and determined that a physiological 1 µL/min was the optimum flow rate to achieve complete mixing. Using Stoke’s Equation and Fick’s second law of diffusion, we determined the amount of time required for the diffusion of commonly used chemotherapeutics across the mixing channel width. At the optimum flow rate, the generated concentration dilution within the device was comparable to the expected concentration dilution. We further determined that the loading procedures were highly reproducible as the median density of the hydrogel-encapsulated cells for each column of wells was similar for all four wells and the cells remained well-distributed throughout the hydrogel. We tested two hydrogel types, a UV-crosslinkable PEGDA and a Michael type addition crosslinkable 4-arm PEG-Ac, indicating that a variety of gelation mechanisms can be accommodated with the device. A drug screening assay was highlighted by generating a dose–response curve for U87 glioblastoma cells treated with temozolomide and carmustine, standard-of-care chemotherapeutics. A limitation of the current device is that it contains a 4 × 4 array of cell culture wells, but an 8 × 8 array could be used going forward to test multiple conditions at the same time and obtain 7 dilutions (including a no drug/soluble molecule control) to build robust dose–response curves. Microfluidic devices, as the one developed here, that incorporate cells, a matrix, and perfusion, are a close mimic of native cellular environments and represent a simple, inexpensive technique that enables multiplexed drug screening.

## Supplementary Information

Supplementary Figures.

## Data Availability

The datasets used and/or analyzed during the current study are available from the corresponding author on reasonable request.
