# Supplementary material for: Hydrogel-based microfluidic device with multiplexed 3D in vitro cell culture
Source: Sci Rep. 2022 Oct 22;12:17781. doi: 10.1038/s41598-022-22439-y (PMC9588086; doi:10.1038/s41598-022-22439-y)
Supplement: Supplementary file 1 — Supplementary Figures. [file 41598_2022_22439_MOESM1_ESM.docx]

**SUPPLEMENTAL DATA**

**For**

**HYDROGEL-BASED MICROFLUIDIC DEVICE WITH MULTIPLEXED 3D *IN VITRO* CELL CULTURE**

^1^Allison Clancy, ^2^Dayi Chen, ^1^Joseph Bruns, ^1^Jahnavi Nadella, ^1^Samuel Stealey, ^2^Yanjia Zhang ^2,^*Aaron Timperman, ^1,^*Silviya P Zustiak

^1^Department of Biomedical Engineering, Saint Louis University, St Louis, MO,

^2^Department of Bioengineering, and Biochemistry and Biophysics, University of Pennsylvania, Philadelphia, PA


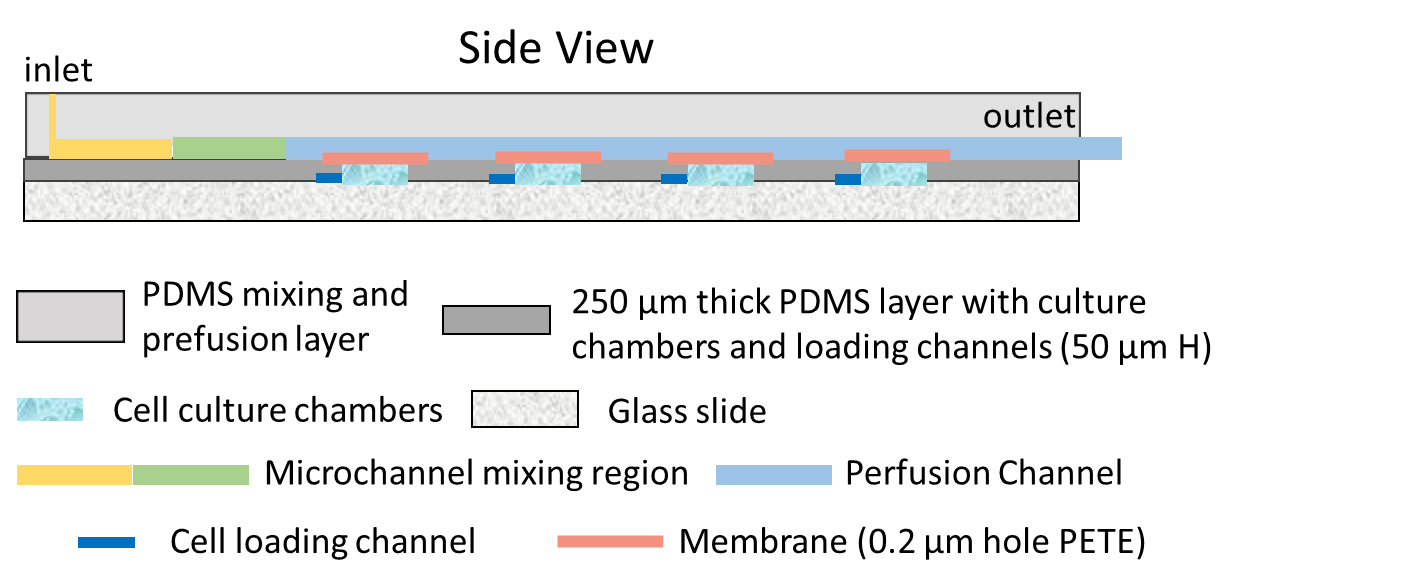


**Supplemental Figure S1:** A detailed schematic of the side view of the fabricated microfluidic device.


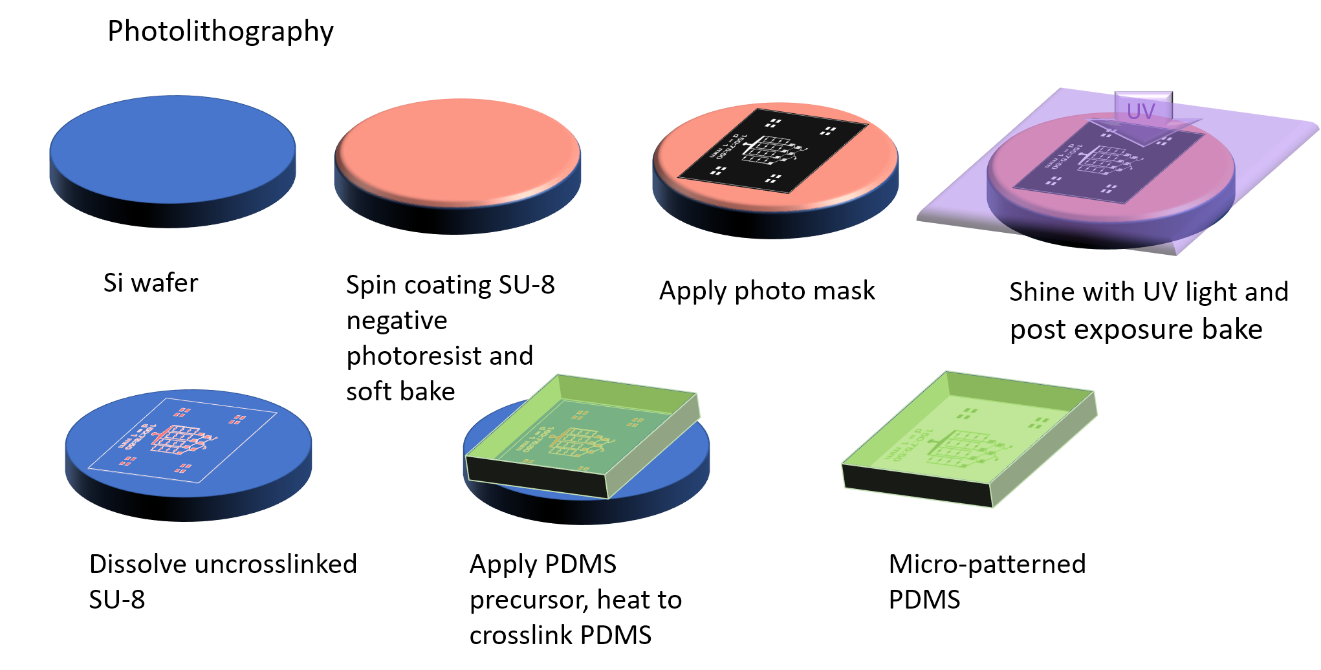


Spin coating SU-8 negative photoresist on Si wafer and soft bake


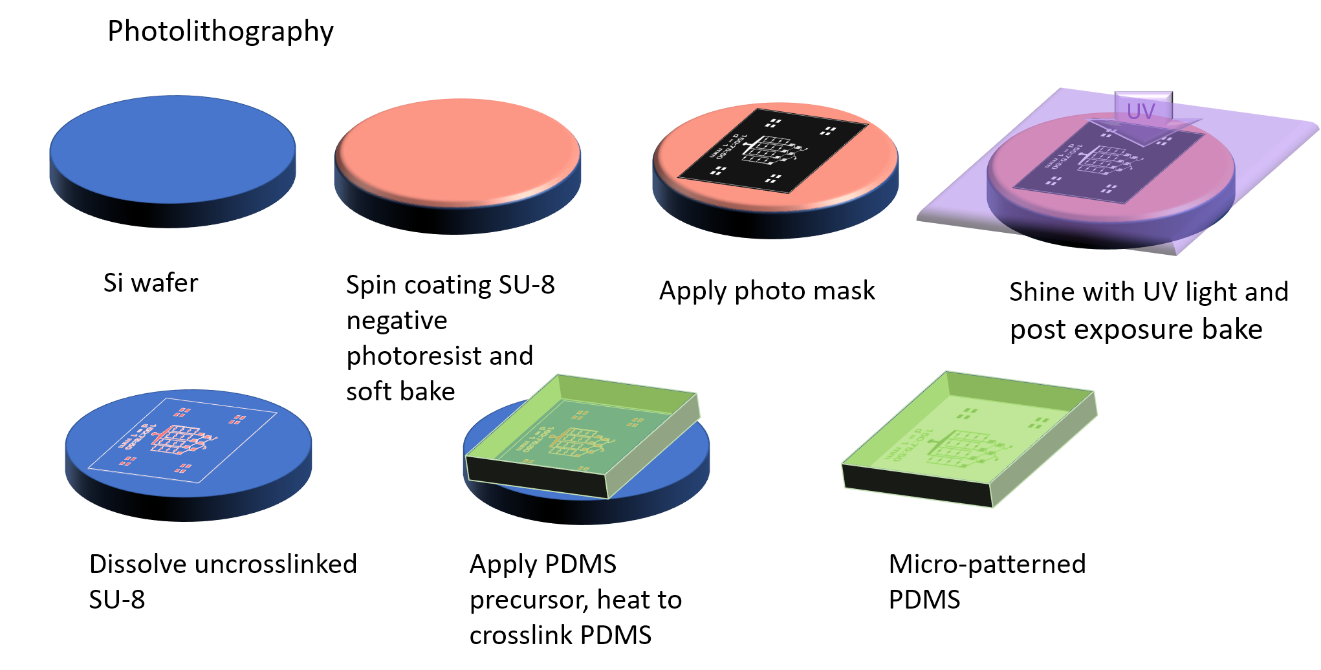


Apply photo mask


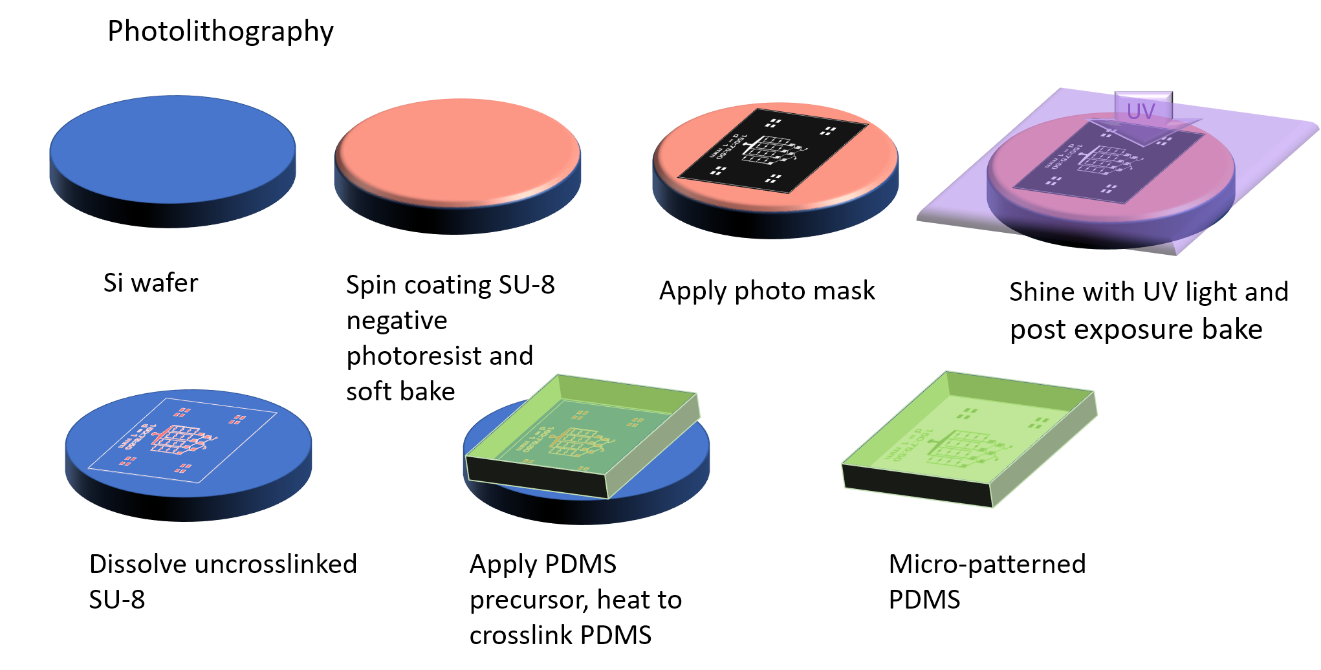


Shine with UV light and post exposure bake


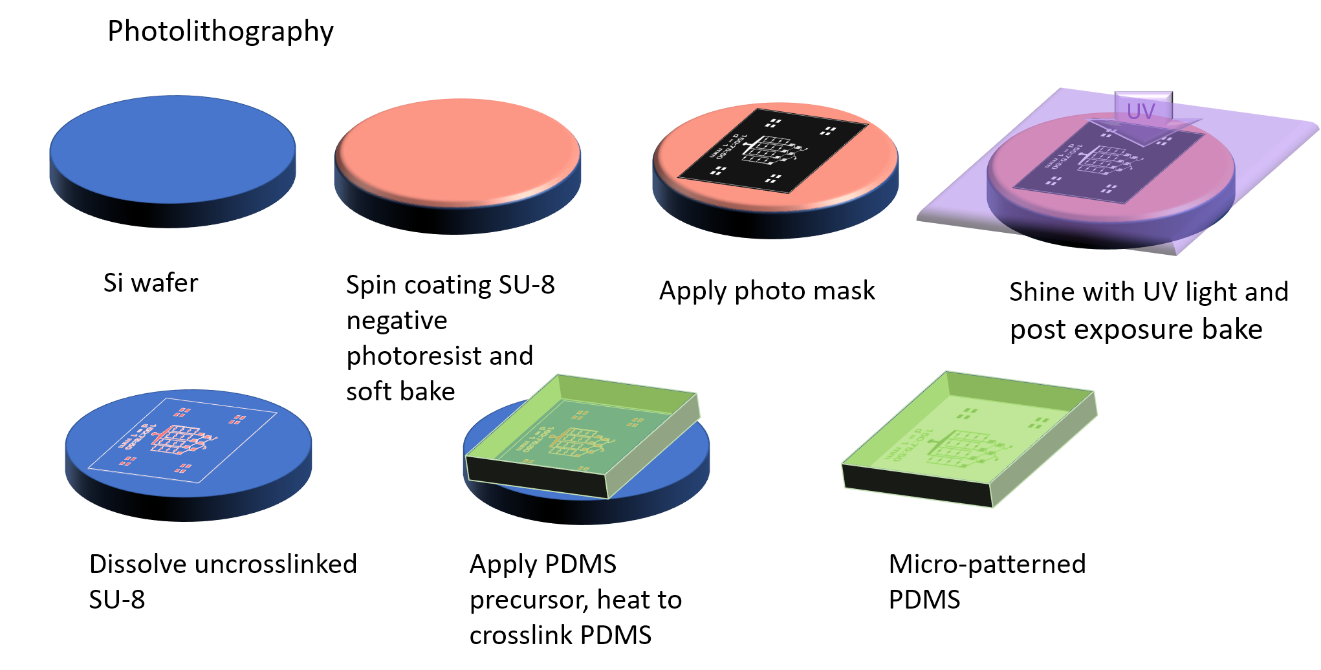


Dissolve uncrosslinked SU-8


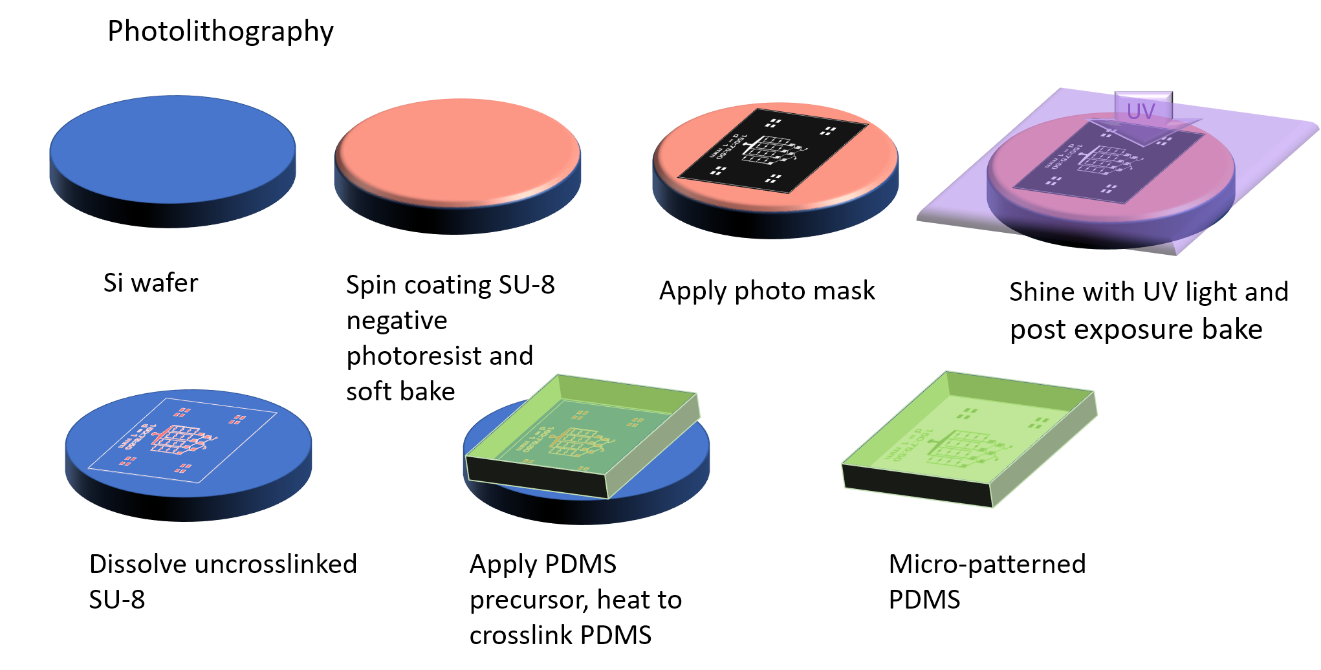


Apply PDMS precursor, heat to crosslink PDMS

x

x


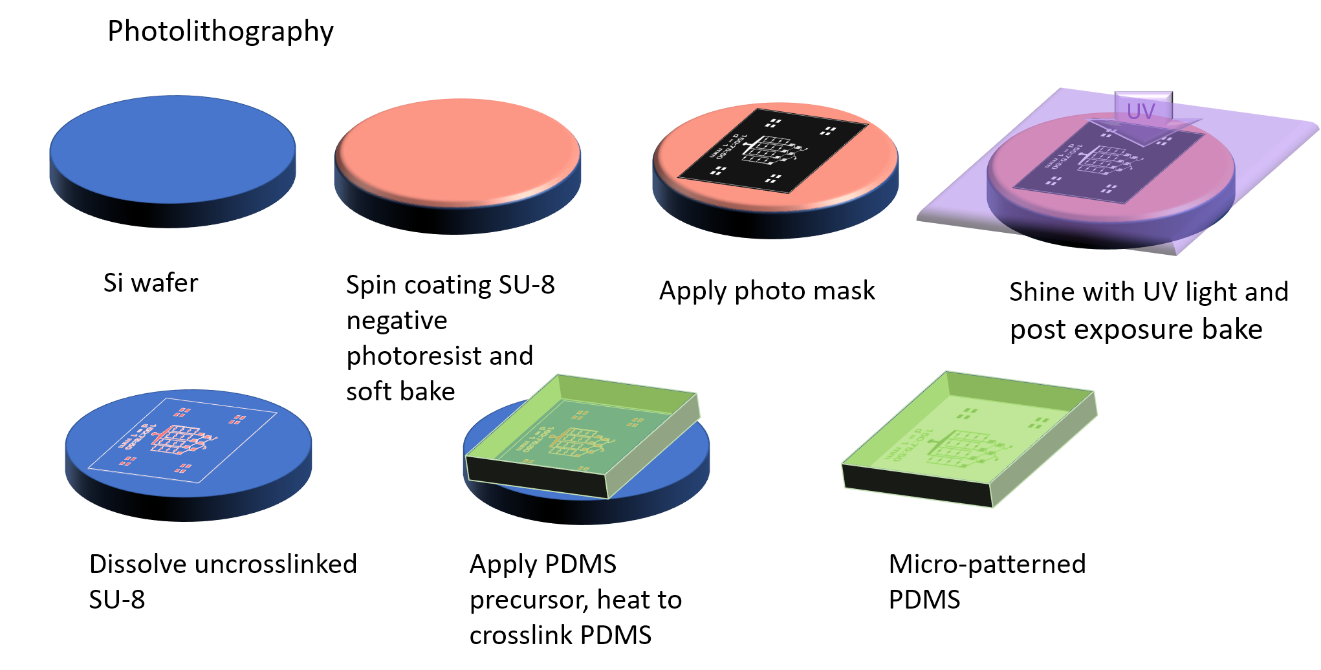


Micro-patterned PDMS

x

x

**Supplemental Figure S2.** **Photolithography process used to create the micro-patterned PDMS layers of the microfluidic device.** In a first step, an SU-8 negative photoresist is spun-coated on a silicon wafer and baked. The photoresist is then illuminated with UV light through a photo mask, baked and dissolved to achieve device mold. PDMS precursor solution is then poured onto the mold and crosslinked to result in a micro-patterned PDMS device.


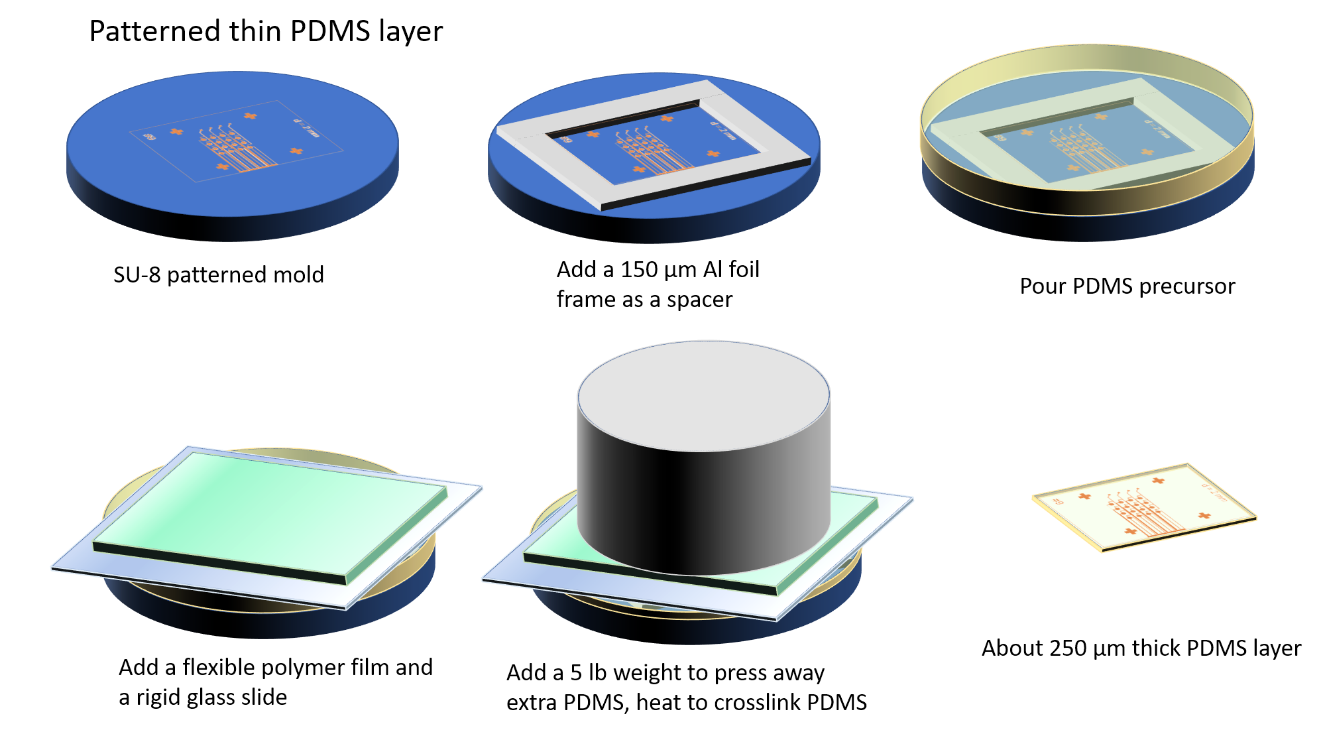


SU-8 patterned mold

Add a 150 𝜇m Al foil frame as a spacer

Pour PDMS precursor

About 250 𝜇m thick PDMS layer

Add a 5 lb weight to press away extra PDMS, heat to crosslink PDMS

Add a flexible polymer film and a rigid glass slide


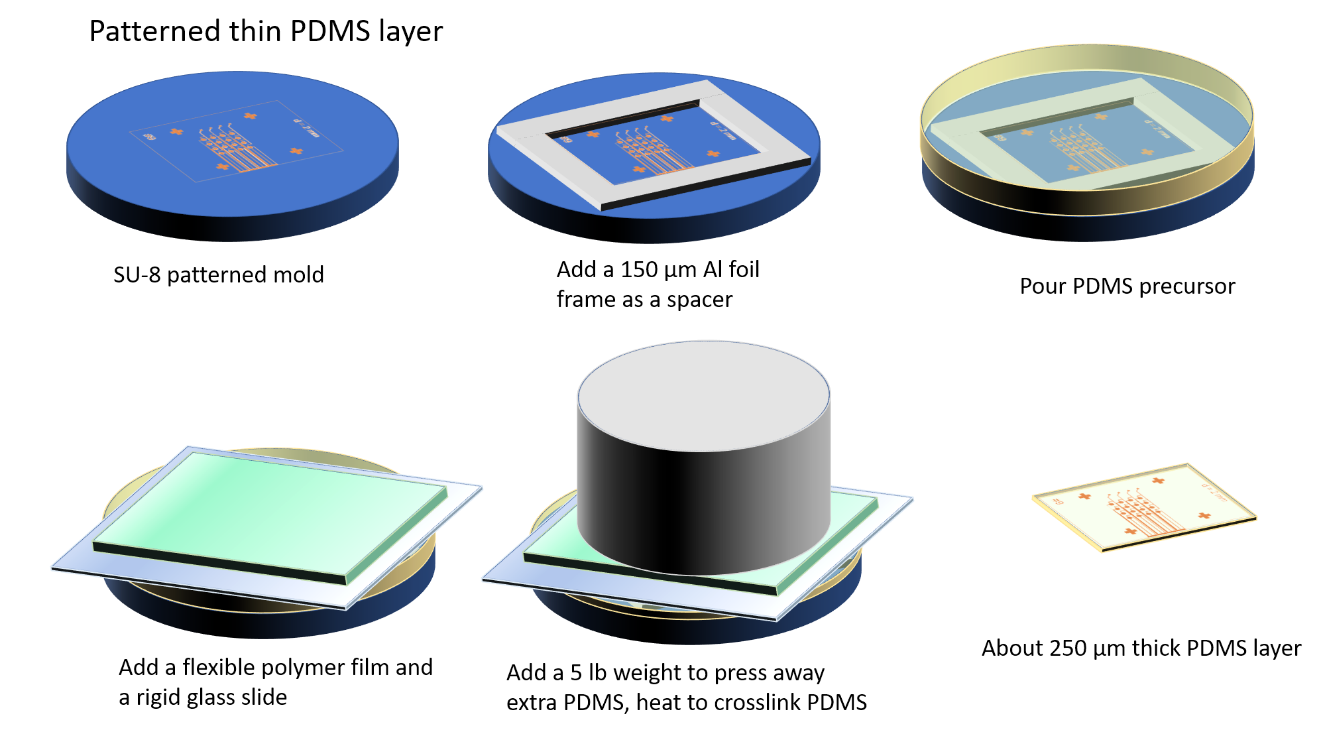


Add a flexible polymer film and a rigid glass slide


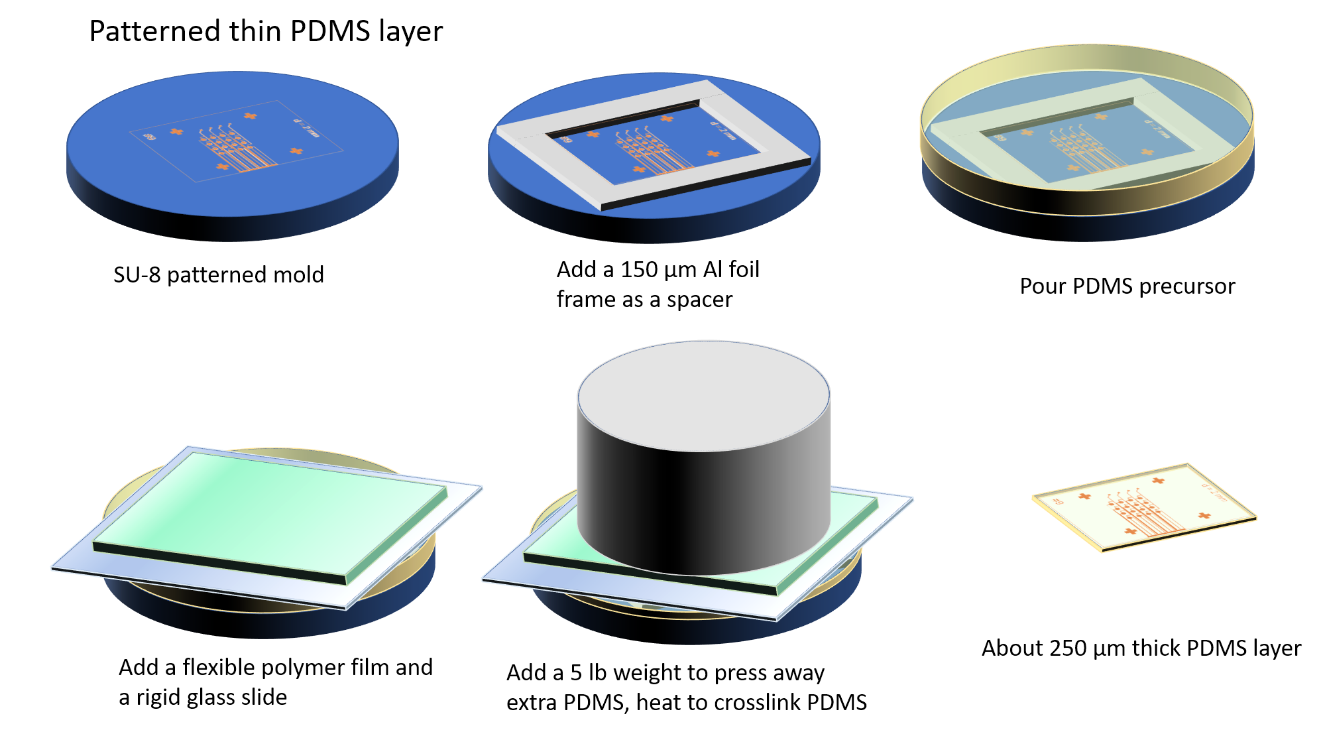


About 250 𝜇m thick PDMS layer


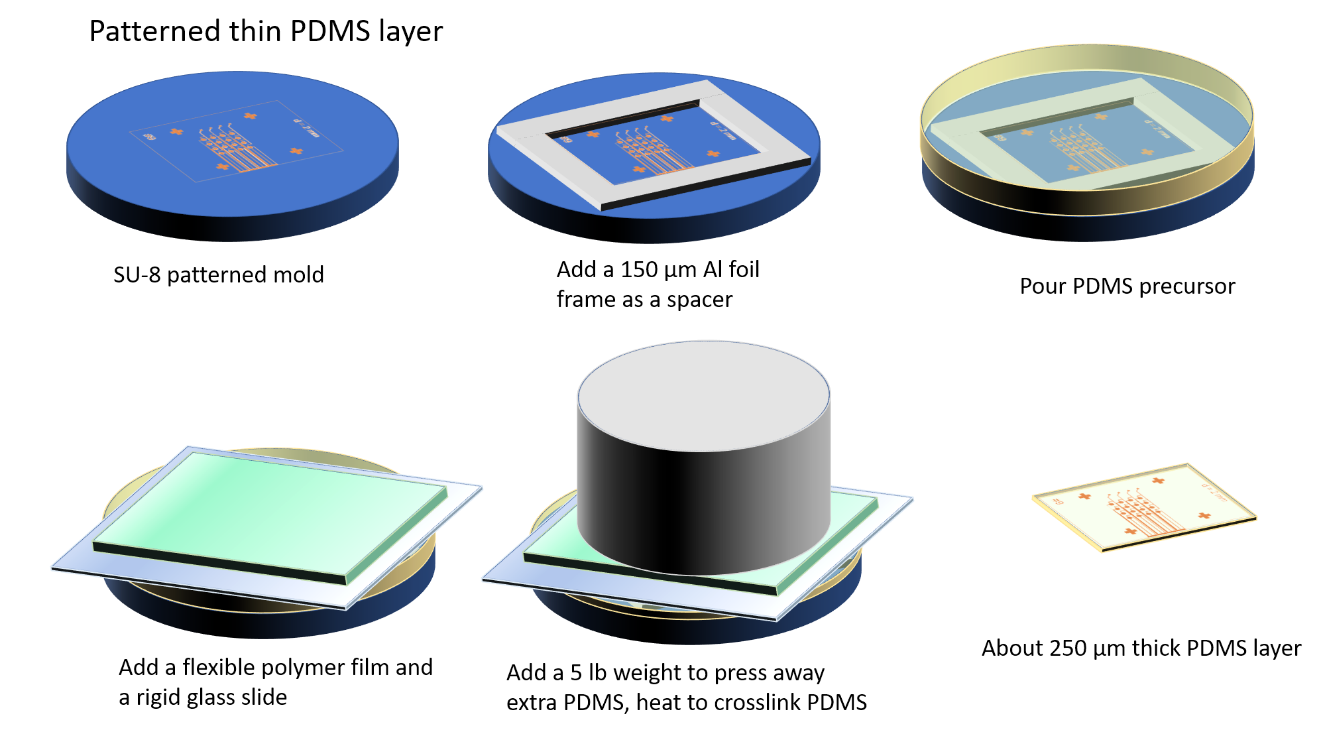


Add a 5 lb weight to press away extra PDMS, heat to crosslink PDMS

**Supplemental Figure S3.** Photolithography process used to create the bottom micro-patterned PDMS layer of the microfluidic device.

**
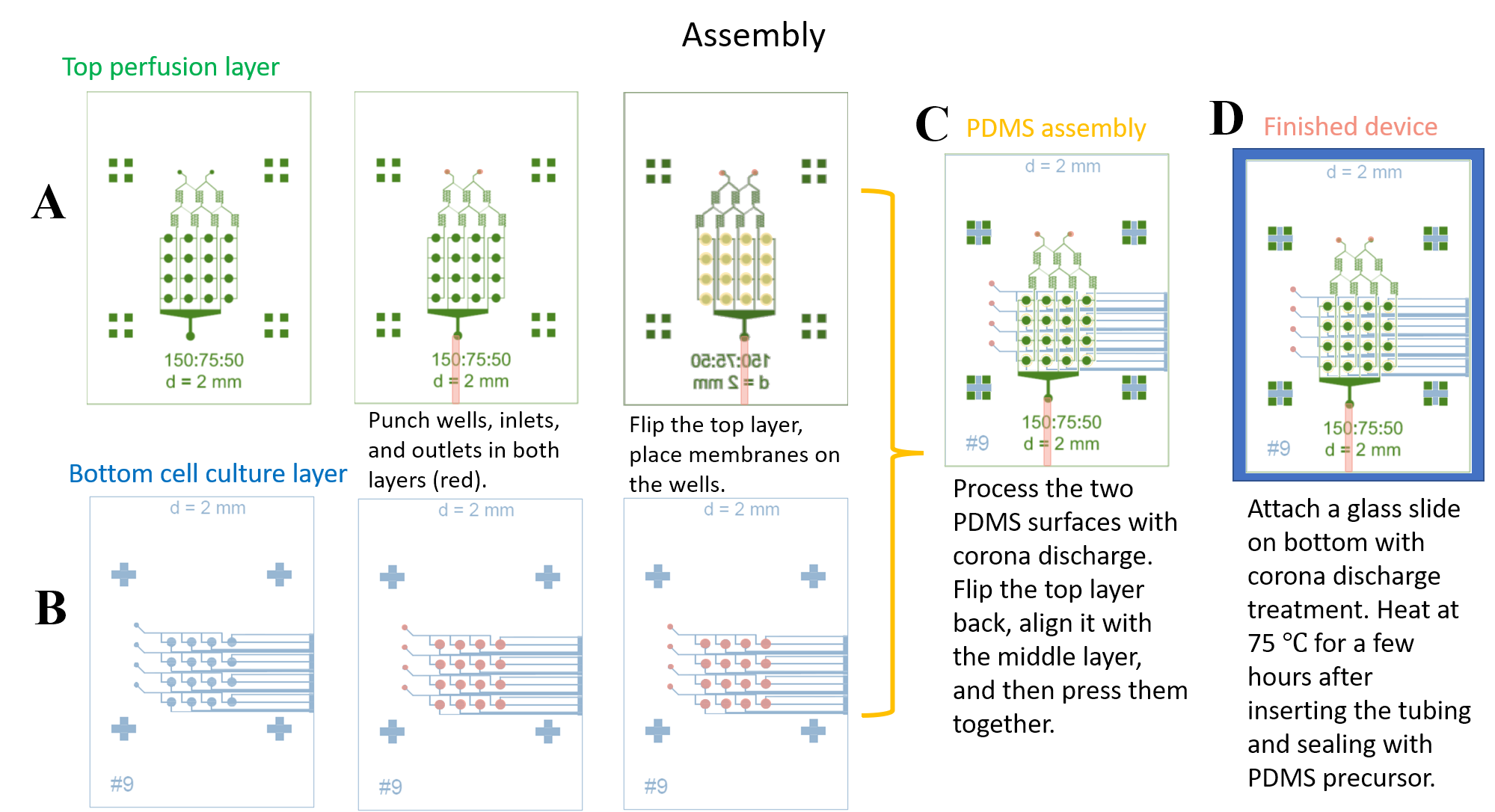
**

**Supplemental Figure S4. Assembling process (step by step from left to right) of the microfluidic devices.** **A)** Assembly of the top perfusion PDMS layer. **B)** The bottom PDMS cell culture layer. **C)** Assembly of the two layers of PDMS. **D)** Assembly of the finished device with a glass slide bottom.


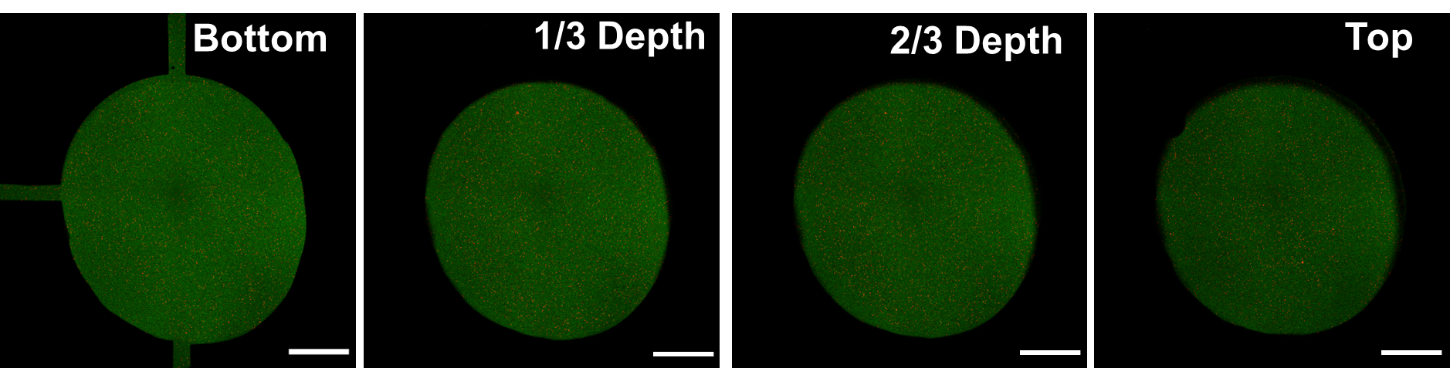


**Supplemental Figure S5**: Images of the bottom, one-third, two-thirds, and the top of a 4-arm PEG-Ac hydrogel when loaded in a cell culture microwell. The hydrogels are labelled with green fluorescence via tethering of FITC-modified ligands to the 4-arm PEG-Ac as described in the methods. Scale bar = 500 µm.


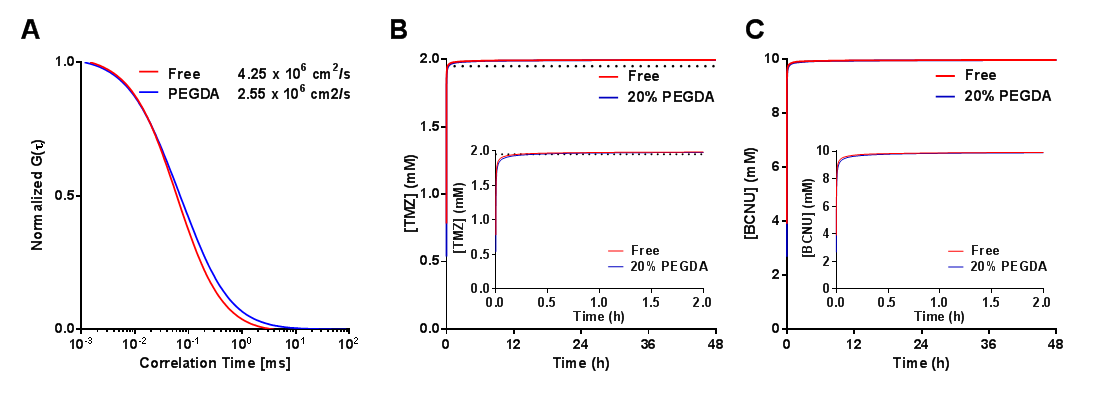


**Supplemental Figure S6: Drug penetration through a PEGDA hydrogel. A**) Normalized autocorrelation curve for *G(t)* for a model fluorescent dye (Atto 655) in a PEGDA hydrogel compared to media only. **B**) Calculated TMZ concentration at full depth of the hydrogel (250 µm). Black dotted line indicates 1.95 mM TMZ. **C**) Calculated BCNU concentration at full depth of the hydrogel (250 µm). The data was modeled using diffusion coefficients at day 1 measured via FCS, using a Fick’s 2^nd^ law and 1-D geometry.


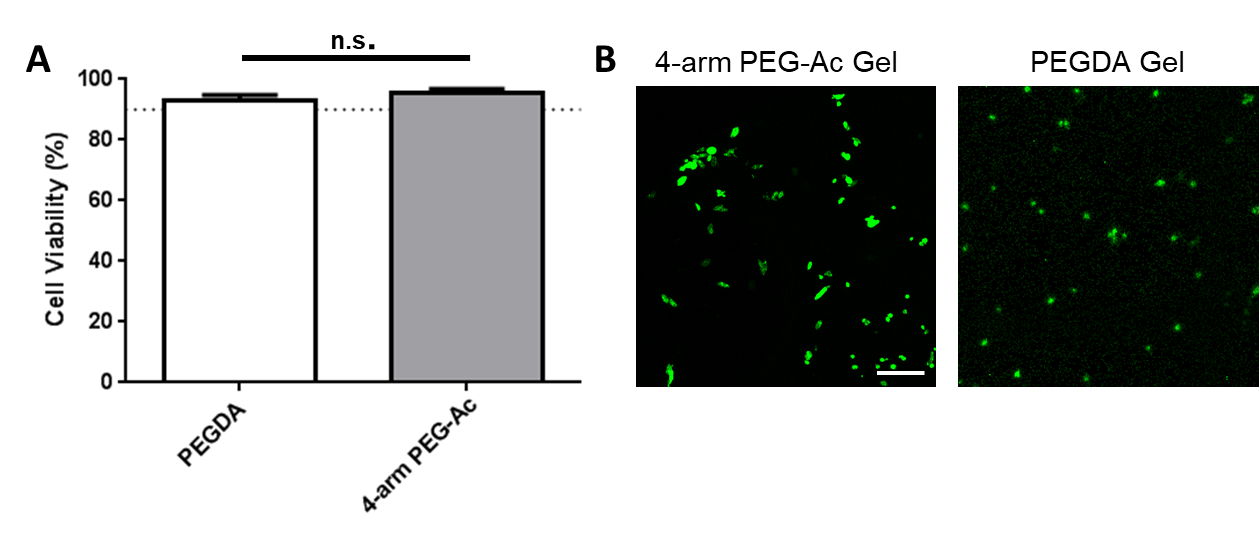


**Supplemental Figure S7:** **A)** Cell viability in 4-arm PEG-Ac and PEGDA is >90% at day 4 of culture (as measured by live/dead staining). **B)** Representative fluorescent images of cell spreading in degradable adhesive 4-arm PEG-Ac hydrogels and cells remaining round in non-degradable, non-adhesive PEGDA hydrogels. Cells were stained with DiOC (green) and cultured in the microfluidic device for 4 days. Scale bar is 100 µm.
